# Temperature-Dependent Swelling of Brodie Graphite Oxide in Liquid Primary Amides

**DOI:** 10.1021/acs.jpcc.6c01991

**Published:** 2026-05-26

**Authors:** Gui Li, Nicolas Boulanger, Bartosz Gurzęda, Christoph Hennig, Kristina Kvashnina, Alexandr V Talyzin

**Affiliations:** † Department of Physics, 8075 Umeå University, Umeå SE-901 87, Sweden; ‡ 55553 Rossendorf Beamline (BOBL-BM20) European Synchrotron Radiation Facility (ESRF), 71 Avenue des Martyrs, Grenoble 38000, France; § Helmholtz-Zentrum Dresden-RossendorfInstitute of Resource EcologyBautzner Landstrasse 400, Dresden 01328, Germany

## Abstract

Swelling in polar solvents is a fundamental property of graphite oxide (GO). Using in situ synchrotron X-ray diffraction (XRD), Brodie graphite oxide (BGO) swelling was studied in a series of primary amides with the number of carbon atoms in the alkyl chain ranging from one (acetamide) to ten (decanamide) and compared to GO swelling in primary alcohols of equivalent chain lengths. The uptake of solvent due to swelling was determined via Differential Scanning Calorimetry (DSC). Swelling of BGO in acetamide, propionamide, and butyramide was found to expand the interlayer distance *d*(001) by ∼3.5–3.7 Å, consistent with the intercalation of a single molecular layer with an orientation parallel to the GO sheets. Swelling in longer molten amides produced larger *c*-lattice expansions correlating with the *c*-unit cell parameter of the pure amides, suggesting two-layer intercalation in a tilted “stand-up” orientation. Reversible swelling transition was found in the BGO–formamide system. This transition corresponds to the change between BGO structures with one-layer and two-layer formamide intercalation and has an enthalpy of 0.01 kJ g^–1^ (BGO). No temperature-driven swelling transitions were observed for the other studied amides, acetamide through decanamide, in contrast to previously reported transitions in BGO–alcohol systems. These results demonstrate the wide tunability of interlayer spacing in BGO–amide systems and highlight the potential of controlled intercalation for applications such as molecular separation and sorption.

## Graphical abstract

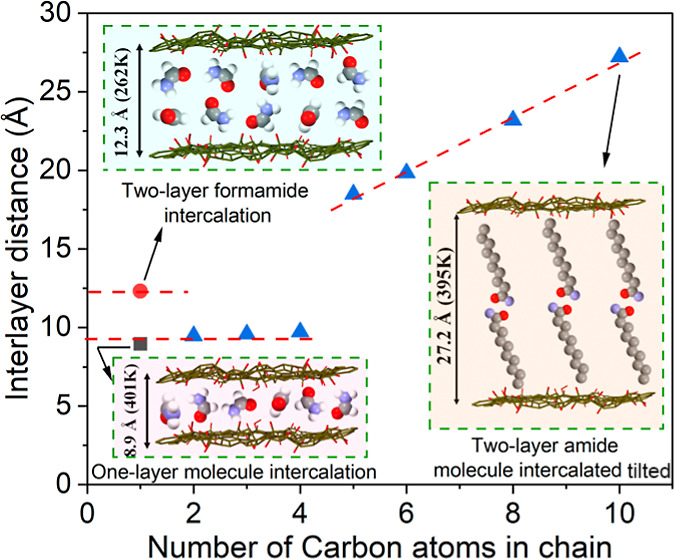

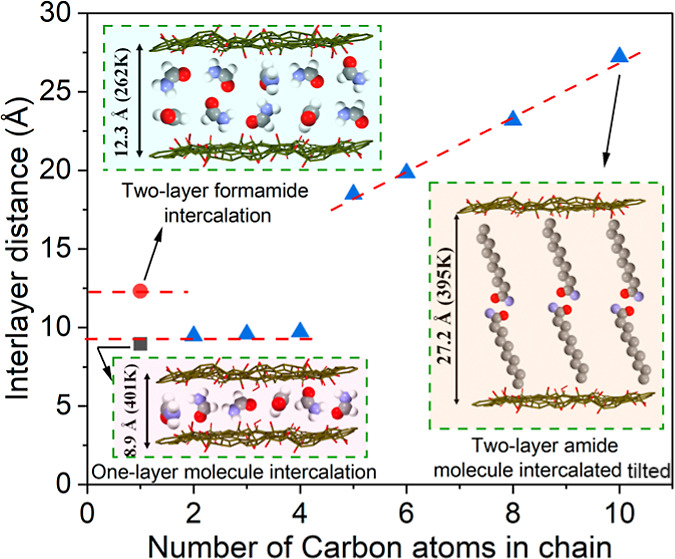

## 1 Introduction

Graphite oxide (GO) is a hydrophilic nonstoichiometric layered material prepared by oxidation of graphite. The most common methods of GO synthesis are the Brodie (BGO) and Hummers methods (BGO).^1,2^ Fuming nitric acid is used in the Brodie method to expand the graphite structure, and potassium chlorate is used for oxidation. The Hummers method relies on concentrated sulfuric acid and potassium permanganate as the oxidant. Many variations have been also reported for the Hummers method, e.g., one of the most common is using mixture of sulfuric and phosphoric acid with a permanganate oxidant.^3–5^ Recently, we also reported a new method combining the oxidant from the Brodie method (KClO_3_) with acids from the modified Hummers’ method.^6^ Other methods also include electrochemical oxidation of graphite in sulfuric acid.^7–10^

It is common for all types of GO that graphene oxide sheets are functionalized with similar types of oxygen-containing groups. Most abundant functional groups are hydroxyls and epoxy, which are attached to the graphene oxide planes, while carboxyl and carbonyl groups are terminating edges or defects of graphene sheets.^11–13^ The *c*-lattice of GO is expanded by 3–4 Å compared to precursor graphite (3.3 Å) due to extensive oxidation. However, the degree of oxidation, type of oxidation, and many other properties of GO depend significantly on the type of the synthesis method and details of synthesis procedures.^13–17^ In fact, GO is not one material but a family of materials with a rather broad variation of properties.^18^

Particularly strong difference is found for GOs prepared using different types of oxidants. In particular, GOs synthesized using chlorates or permanganate show a strong difference in types of oxygen functional groups and their relative abundance, number of defects, thermal exfoliation temperature, swelling in polar solvents, etc.^6,14,16,17,19–21^

GO is hydrophilic and can be easily dispersed in water or other polar solvents. The GO dispersions are commonly used to prepare multilayered graphene oxide membranes suggested for various applications such as separation of gas mixtures, water purification, desalination, and energy storage.^22–28^ Swelling is one of the most important properties of GO, enabling these applications, and studied for over 100 years.^9,29–31^ Polar solvents, such as water or alcohols, intercalate between the individual GO layers, significantly increasing the interlayer distance and creating solvent filled “permeation channels” in membranes.^32,33^ It should also be noted that swelling of graphene oxide membranes prepared from aqueous dispersions is distinctly different compared to that of precursor GO,^33^ mostly due to the effects of rapid air aging.^34^

Extensive studies of GO swelling in water, alcohols, and several other polar solvents have been performed over the past three decades.^35–41^ It is well-known by now that swelling properties of GOs depend very strongly on the synthesis method.^16,42–44^ The first studies of GO swelling in alcohols with long alkyl chains were performed in the 1960s,^45^ suggesting different possible types of geometrical arrangement for intercalated molecules in 1-alcohols (parallel and perpendicular to graphene oxide sheets) depending on the length of molecules and temperature.^46,47^ It should be noted that some of these early reports do not provide common (for modern times) characterization of GO (e.g., degree of oxidation, FTIR, etc.) and are difficult to compare with more recent studies.

Our group performed systematic studies of GO swelling in various polar solvents as a function of pressure and temperature over the past 15 years, revealing many effects.^18^ Possibly the most interesting are swelling transitions found for BGO in several polar solvents. These transitions were found first in high pressure experiments with GO immersed in water and alcohols.^48–50^ Later, similar swelling transitions were found also in temperature-dependent structural studies with a broad range of polar solvents,^42–44,51–53^ which were related to changes in the amount of solvent inserted between GO flakes. The most common type of swelling transitions have been found for BGO in, e.g., methanol, acetonitrile, acetone, and dimethylformamide and corresponds to a change between one-layer and two-layer intercalation of solvent molecules.^42–44,54^ This type of transition is characterized by a step-like increase of interlayer distance upon cooling of BGO in excess liquid solvent and a distinct anomaly detected by Differential Scanning Calorimetry (DSC). The changes of interlayer distance and the composition of the solid solvate phase have been found to correspond to insertion of an additional solvent layer at low temperature and reversible deinsertion of this layer upon heating.^6,43,55^ Similar swelling transitions related to insertion and deinsertion of a single solvent layer have also been reported for several 1-alcohols that form a solvate phase with several solvent layers intercalated between GO sheets. For example, transitions between the four-layer solvate phase and five-layer solvate phase have been found for BGO in octanol.^54^ Remarkable intercalation of several alcohols in the BGO structure with the formation of a precise number of molecular layers (layered intercalation) have been confirmed by observing layer-by-layer evaporation of solvent using XRD and thermogravimetric analysis (TGA).^21,56^ The swelling transitions related to insertion/deinsertion of the solvent layer have been found so far only for BGO. The swelling transitions related to insertion/desertion of one layer of solvate molecules between GO sheets have been defined in our earlier study as “Type I”.^44^ The Type I swelling transitions have been found so far only in BGO, e.g., immersed in liquid methanol, ethanol, propanol, heptanol, octanol, and nonanol. In contrast, no swelling transitions have been found for BGO in butanol, pentanol, and hexanol.^44^

Inhomogeneous oxidation of HGO and interstratification of differently hydrated/solvated interlayers are believed to be the main reason for the absence of sharp swelling transitions and strong but gradual temperature-dependent changes of *d*(001) observed by XRD for HGO immersed in the same solvents.^17,42,57^

Recently, systematic studies of BGO and HGO swelling have been performed in our group for a set of 1-alcohols with the chain length starting from 1 carbon atom (methanol) and up to 22 carbons (1-docosanol).^42,44,58^ Both BGO and HGO immersed in liquid 1-alcohols just above the solvent’s melting points showed distinctly different types of solvent intercalation for molecules with the number of carbon atoms in the molecule chain below and above ten. Layered intercalation of alcohol molecules with a parallel to GO sheets orientation was found in shorter 1-alcohols, while longer alcohols form densely packed two-layer structures with the orientation of molecules perpendicular to the GO sheets. Both BGO and HGO exhibited another type of swelling transition as a function of temperature in alcohols with the carbon chain longer than ten atoms. This swelling transition (named as Type II) corresponds to a change in the orientation of intercalated solvent molecules from perpendicular to the GO plane (in the low-temperature phase) to parallel to the GO plane in the high-temperature phase.^42,44^ The change in the orientation of intercalated molecules relative to graphene oxide layers from “standing up” to “lying down” has been suggested already in some early studies.^31,47,59^ Our study of HGO and BGO swelling in longer alcohols revealed a strong decrease in the amount of intercalated solvents at the point of the Type II swelling transition, correlating with the change of the molecular orientation from perpendicular to GO layers at lower temperature to parallel to GO planes at higher temperature.^42,44^

It should be noted that both Type I and Type II swelling transitions should be described as incongruent melting of the low-temperature phase with a partial release of solvent from the solid phase into the liquid phase.^58^ So far, Type II swelling transitions have been found only in GO systems with long alcohols, while systematic studies of swelling on other chain molecules are not available. Therefore, it is interesting to verify if similar trends in temperature-dependent swelling of GO can be found in other polar solvents with hydrophilic end groups and progressively longer alkyl chains.

Alkyl amides are common polar solvents that have never been studied for GO swelling.^60^ So far, the swelling of BGO has been studied only in one amide solvent, dimethylformamide (DMF). Intercalation of DMF into the BGO structure was found to be single-layered at ambient temperature with a Type I transition into a two-layered structure upon cooling.^43^ Therefore, it is logical to expect that GO in short primary amides is also likely to exhibit swelling transitions of Type I. Moreover, since the structure of primary amides includes the same alkyl chains as 1-alcohols, it could be suggested that some trends in swelling will also be similar for primary amides and alcohols. Possible differences in swelling of GO in these two groups of solvents would be then assigned to the properties of the hydrophilic end group.

In this study, we report temperature-dependent swelling of BGO in a set of primary amides including formamide, acetamide, propionamide, butyramide, valeramide, hexanamide, octanamide, and decanamide and compare it to swelling of BGO in 1-alcohols. In situ synchrotron XRD and DSC were used to study the structure and swelling transitions in these systems. A Type I swelling transition between one-layer and two-layer structures was found for BGO in formamide but not in other amides. The trends in swelling of BGO in amides with progressively longer alkyl chains were analyzed and compared to swelling in alcohols. The difference in swelling properties of BGO in alcohols and amides is assigned to stronger amide–amide group interactions, which hinder effects related to incongruent melting of solid solvates.

## 2 Experimental Methods

BGO was synthesized using a slightly modified Brodie method with a single oxidation step, as described in our earlier studies. Moreover, we used the material from the same batch that was characterized in detail in our previous publications.^44,61^ Characterization of this batch was performed using XRD, XPS, FTIR, and TGA (see the Supporting Information). The BGO material showed a C/O ratio of 2.65 according to XPS. Figure S1 presents the XRD pattern recorded from BGO under dynamic vacuum conditions with an interlayer distance of 6.0 Å.

Formamide (99.5%), butyramide (98%), pentanamide (97%), hexanamide (≥98%), and decanamide (≥98%) were purchased from Fisher Scientific. Acetamide (99%), propionamide (97%), and octanamide (≥98%) were from VWR company.

DSC scans of BGO in different amides were recorded in gold-plated aluminum or aluminum DSC capsules by a STARe system 3 by Mettler Toledo under a nitrogen flow. All of the samples were studied for three cycles of heating and cooling with a temperature change rate of 1 K/min. The values of sorption and enthalpy were calculated based on the second and third cycles. The main error in the results is due to the balance accuracy (0.1 mg). The weight ratio of BGO to amide used for XRD and DSC experiments was selected to ensure an excess of solvent sufficient for saturated swelling. Table S1 lists the weights of BGO and amides used for DSC measurements.

In situ XRD experiments with heating and cooling were carried out at the Rossendorf Beamline (BM20), ESRF, using a Cryostream 8000System (Oxford Cryosystem) for temperature controlling. The X-ray wavelength was 0.727692 Å.

BGO and each amide (except for formamide, which is liquid at ambient temperature) were first ground together and then loaded into a 1 mm diameter glass capillary 2 weeks before the synchrotron visit. Formamide was added to the capillary directly after loading the BGO powder. A large excess of solvent was used in each experiment to ensure saturated swelling.

In our in situ experiments, samples were heated rapidly above the melting point of each amide, and then, temperature was increased to 463 K at a controlled speed (4 K/min). The temperature was kept stable at 463 K for 5–10 min and, if no swelling transition was found, rapidly cooled back to ambient temperature at a cooling rate of 30 K/min. For BGO in formamide, slow cooling was used to verify reversibility of the swelling transition (4 K/min). All the data were collected in transmission geometry using a DECTRIS PILATUS3 × 2 m Si area detector and extracted with Bubble software. Before each temperature change procedure, 2–5 XRD patterns at room temperature were obtained as references. Afterward, XRD data were recorded continuously with 10 s exposure time during the heating and cooling processes. Filters were implied to adjust the intensity of the X-ray beam to avoid sample damage.

## 3 Results

BGO swelling in liquid primary amides was studied here as a function of temperature using in situ synchrotron radiation XRD and DSC. The study was mostly focused on the temperature interval above the melting point of amides and up to 463 K, not exceeding this temperature to avoid thermal degradation of GO. The set of amides used in this study includes molecules with the number of carbon atoms ranging from 1 (formamide) to 10 (decanamide). In the following, the amides are named according to the number of carbon atoms in the solvent chain: e.g., C10 for decanamide. Only formamide is liquid at ambient temperature, while all other amines studied here are solids with melting points in the temperature range ∼350–380 K (Table 1).

**Table 1:** Summary of Structural Data of BGO in Amides^b^

System	Melting point [K] ^63–65^	*d*(001) [Å]	Δ*d*[Å] (Swelling)	Composition [g/g]	Composition [mol/g]
BGO–C1 (2L)	275	12.32	6.32	0.67 (±0.01)	0.0150
BGO–C1 (1L)		8.97	2.97	0.33^a^	0.0073
BGO–C2	374	9.48	3.48	0.73 (±0.04)	0.0124
BGO–C3	353	9.59	3.59	0.89 (±0.07)	0.0122
BGO–C4	387	9.72	3.72	1.14 (±0.09)	0.0130
BGO–C5	377	18.48	12.48	1.18 (±0.08)	0.0116
BGO–C6	373	19.83	13.83	1.33 (±0.1)	0.0115
BGO–C8	378	23.20	17.20	1.45 (±0.1)	0.0101
BGO–C10	371	27.22	21.22	1.61 (±0.1)	0.0094

a The value is taken as half of sorption determined using DSC for 2L phase.

b From left to right: melting point of pure amide, *d*(001) value, recorded just after the complete melting of amide and saturated intercalation into the BGO structure; interlayer distance change due to swelling relative to the vacuum dry value of BGO *d*(001) = 6 Å; sorption of amides in g/g (solvent/BGO) and mol/g (solvent/BGO) just above the melting point of amide determined using the DSC method.

In situ XRD experiments performed over the temperature range from the melting point of amide up to 463 K revealed that BGO exhibits swelling in all C1–C10 amides studied here. However, a distinct swelling transition was observed only for BGO in liquid formamide within studied temperature intervals. Below, we first present the results obtained for the BGO–formamide system, where a swelling transition is evident, and continue with an analysis of BGO swelling as a function of alkyl-chain length across the full series of amides.

### 3.1 Swelling Transition in the BGO–Formamide (BGO–C1) System

Figure 1 shows XRD patterns recorded from the BGO–formamide system during heating and cooling, respectively. Formamide (C1) is liquid at room temperature. Therefore, the sample of BGO immersed into excess liquid formamide was first cooled below the point of solidification, and next, the temperature increased from 230 to 423 K, followed by second cooling down to 273 K. As expected, the XRD patterns recorded at temperatures below the point of solvent solidification exhibit reflections from BGO and solid C1 (Figure 1a). The reflections from solid C1 disappear above the melting point of solvent starting from 262 K. Swelling of BGO in liquid formamide results in expansion of the *c*-lattice compared to the solvent-free state. Analysis of XRD data also shows evidence for a sharp Type I swelling transition between the two BGO–C1 phases (Figure 1c). The low-temperature phase (α-phase) shows a maximum *d*(001) value of 12.3 Å, which is about 6.3 Å larger than that of the solvent-free BGO structure of *d*(001) = 6.0 Å recorded under dynamic vacuum conditions (Figure S1). The *d*(001) of the high-temperature phase (β-phase) is 8.9–9.0 Å. The difference between *d*(001) values calculated for low-temperature and high-temperature phases is about 3.3 Å, which is in a good agreement with the size of the formamide molecule.^62^ The thickness of the first intercalated layer is then about 3.0 Å, and the thickness of the second layer is about 3.3 Å. The solvent layers are completely disordered as it follows from the absence of reflections other than those of BGO. Figure 1d shows a schematic illustration for the structures of the low-temperature and high-temperature phases of BGO–C1.

**Figure 1:**
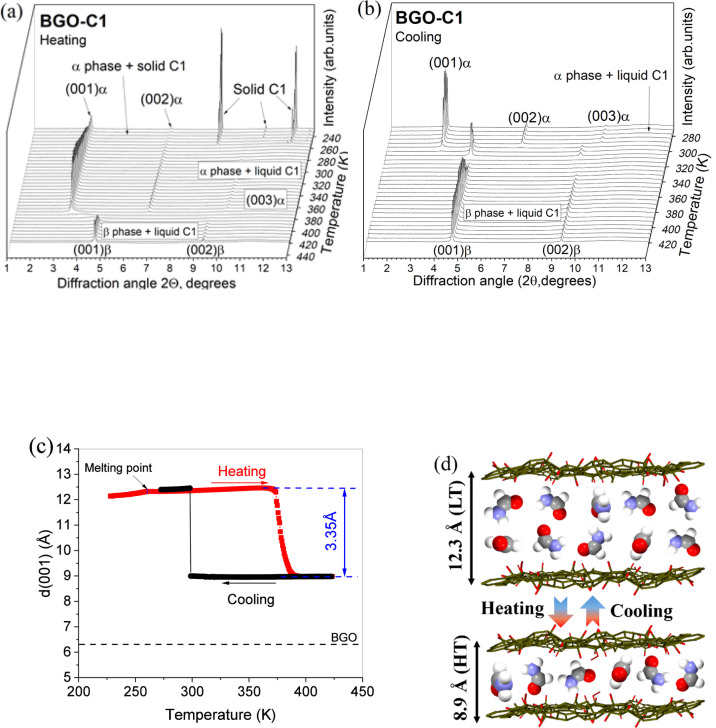
XRD patterns of BGO immersed in molten C1 recorded in situ during (a) heating and (b) cooling at a temperature change rate of 4 K/min. (c) Temperature dependence of *d*(001) recorded from the BGO–formamide sample. (d) Schematic illustration of C1 intercalated between BGO sheets at low temperature and high temperature (λ = 0.727692 Å).

The swelling transition found in the BGO–C1 system is similar to swelling transitions previously observed for BGO immersed in several other polar solvents, e.g., methanol, ethanol, and acetonitrile.^6,43^ The similarity includes the change from single-layer intercalation at higher temperatures to two-layer intercalation at lower temperature and swelling-induced ordering reflected in sharper peaks of the low-temperature phase, the larger number of reflections in the (00l) set, and reversibility of the swelling transition.

Notably, relatively strong hysteresis is found in the heating–cooling runs, indicating relatively strong effects of overheating and overcooling. The reversible swelling transition is also confirmed using DSC scans performed with liquid immersed BGO samples (Figure 2). The sample of formamide shows only anomalies due to melting and freezing, while the scans recorded from the 1:1.12 g/g (BGO/C1) sample exhibited additional anomalies. The heating half cycle shows anomaly due to incongruent melting of the low temperate two-layered (2L) BGO–C1 phase corresponding to the removal of one C1 layer at ∼349 K during the heating. Reverse transition corresponding to the transition from the 1L- to 2L-phase is found in the cooling half cycle scan at around 278 K. The enthalpy of swelling transition of 0.01 kJ/g (BGO) is in agreement with earlier results obtained for similar 1L- to 2L-phase swelling transitions in other common polar solvents (e.g., ∼0.01 kJ/g for the BGO–methanol system).^43^

**Figure 2:**
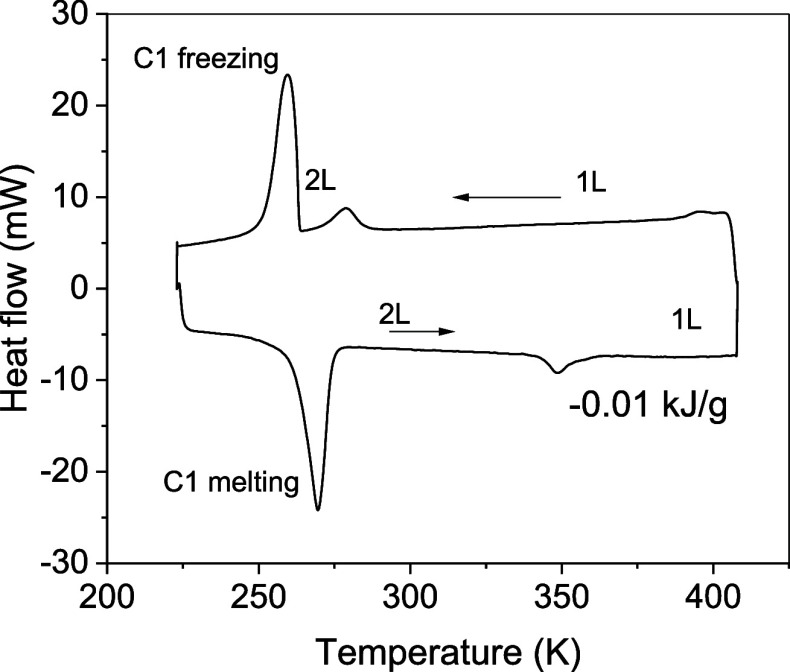
DSC scans recorded from sample BGO–formamide (1:1.12) during heating and cooling.

Therefore, our experiments provide the first observation of a swelling transition in BGO–alkyl amide systems. The Type I swelling transition found in this system can be confidently assigned to the change of the BGO structure from the two-layer solvent intercalated phase (2L-phase) at low temperatures to one-layer (1L-phase) intercalated at high temperatures.

### 3.2 Swelling of BGO in Alkyl Amides Other Than Formamide

Except for formamide, all other alkyl amides are solid at ambient temperature. The temperature dependence of *d*(001) within the temperature interval between the melting point of amides and 463 K shows only minor continuous shifts and no sharp anomalies due to swelling transitions (Figure 3). The continuous shift of the *d*(001) value is the strongest for BGO in C10 (∼1 Å), but it is still much smaller than the size of the amine molecule. Experiments above 463 K were not performed to avoid possible issues related to thermal degradation of BGO. It should be noted that BGO has higher thermal stability than common HGO.^6,17^ The TGA data recorded from the same batches of HGO and BGO have been analyzed in our earlier studies. While HGO shows a significant gradual weight loss above 413 K and up to the major thermal deoxygenation step (∼463 K), BGO shows only a rather minor weight loss up to ∼480 K.^6,44^ Additional experiments performed using TGA at 462 K showed an overall weight loss of only ∼0.6% over the period of 2 h (Figure S16 in the Supporting Information file), confirming the thermal stability of BGO within the time intervals of experiments with swelling in liquid amines (Figure 3). The temperature dependence of *d*(001) is almost flat for BGO immersed in all tested amides, thus also confirming the absence of thermal degradation.

**Figure 3:**
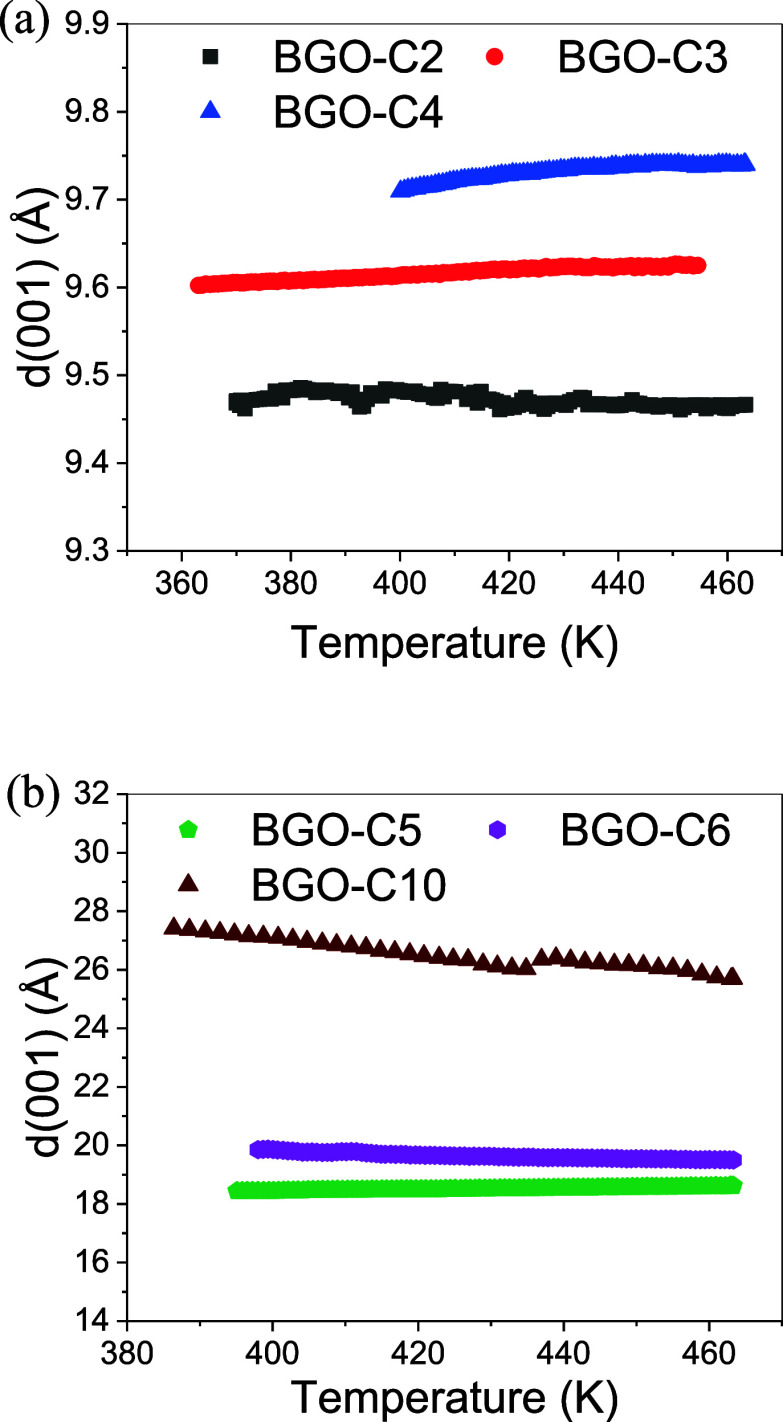
Temperature dependence of *d*(001) from BGO in (a) C2–C4 and (b) C5, C6, and C10 during heating (λ = 0.727692 Å). The data were recorded during heating with a 4 K/min ramp.

The detailed plots showing the evolution of XRD patterns recorded in each system as a function of temperature are provided in the Supporting Information (Figures S2–S7).

Below, we discuss in more detail how the swelling of BGO depends on the length of the alkyl chains. The swelling of BGO is the easiest to compare by using the value of *d*(001) recorded just above the point of solvent melting. The *d*(001) provides information about *c*-lattice expansion due to intercalation of solvent and the composition of this solvate phase determined using the DSC method (Table 1).

XRD patterns of BGO recorded in liquid amides just above the temperature point of melting are presented in Figure 4a. BGO demonstrates swelling in all molten amides studied here. It should be noted that this result is not as trivial as it might look. It is known that GO swells in polar solvents and does not swell in nonpolar. The molecules studied here consist of a hydrophilic amide group and an alkyl chain, which is hydrophobic. The longer the alkyl chain is, the less hydrophilic the molecule is. The amides with an alkyl chain longer than 5 carbon atoms are not miscible with water. Nevertheless, swelling of BGO is observed in both long-chain alcohols studied earlier and, as revealed in experiments presented here also, long-chain amides up to C10 in this study. However, the *c*-lattice expansion due to the swelling in amides is strongly dependent on the length of the alkyl chain and shows a nontrivial trend.

**Figure 4:**
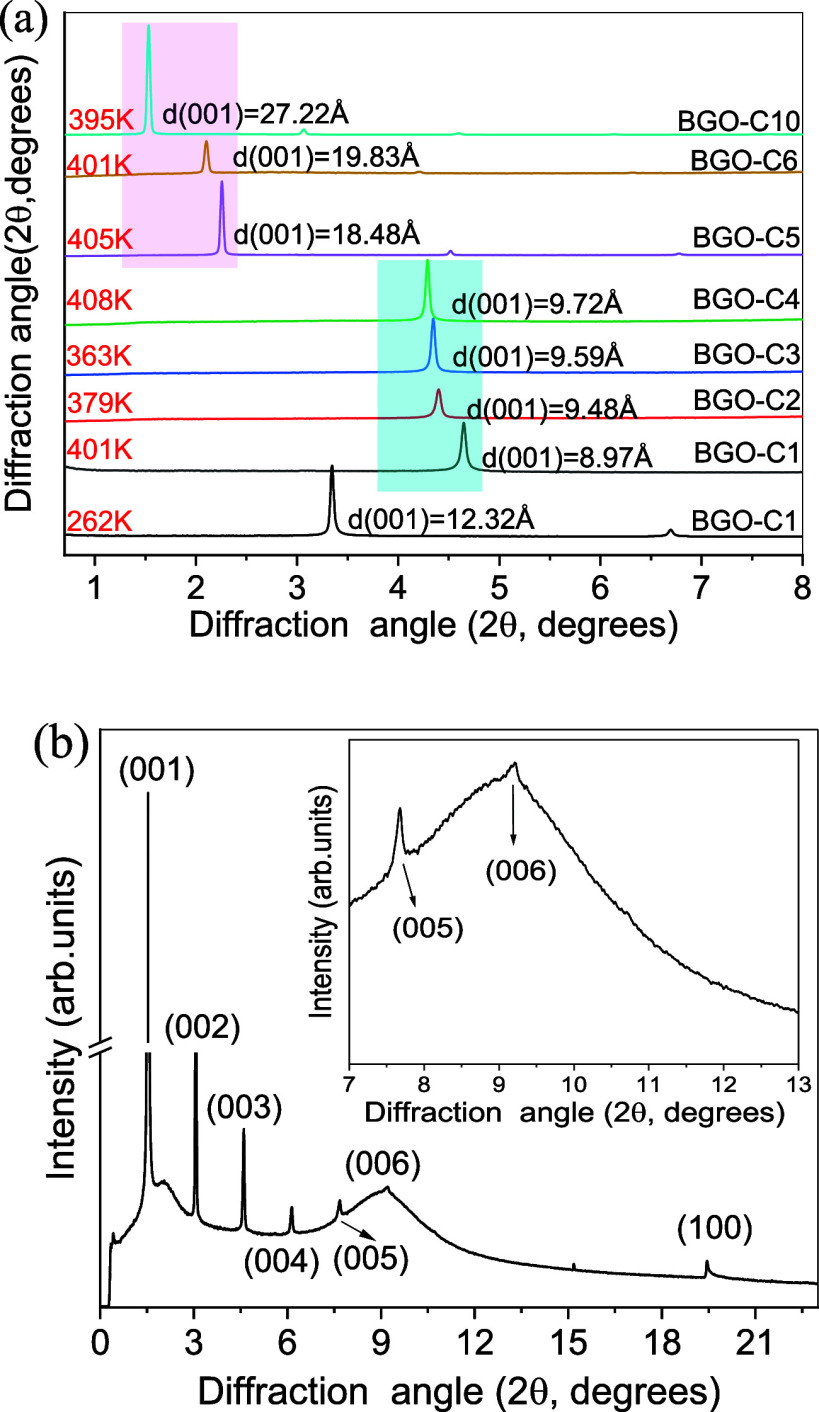
(a) XRD patterns of BGO recorded in liquid amides at specified temperatures (λ = 0.727692 Å). For BGO–C1, XRD patterns of both low-temperature and high-temperature phases are shown; for other BGO amides, the patterns recorded just above the melting point of the solvent are selected. (b) XRD pattern of BGO–C10 after the melting of decanamide shown in broader range of diffraction angles.

As previously discussed, BGO in liquid C1 exhibited two swollen phases. Therefore, Figure 4a includes XRD patterns recorded from both phases, the first one with an interlayer distance of 12.32 Å (262 K) and the second one with *d*(001) = 8.97 Å (401 K). All XRD patterns recorded from BGO in molten amides showed no additional reflections due to intercalated solvent. As an example, Figure 4b shows the XRD pattern of BGO recorded in molten decanamide (C10). The pattern shows set of (00l) reflections with order up to 6, (100) reflection, and broad feature from liquid solvent.

Analysis of data shown in Figure 4 and Table 1 allows us to distinguish two distinctly different groups. The interlayer spacings of BGO in molten C2, C3, and C4 are found to be in the 9.5–9.7 Å range, pointing to similar structures of BGO–C2, BGO–C3, and BGO–C4 phases. The structure of these phases is expanded by approximately 3.2–3.4 Å relative to pure BGO. This value of *c*-lattice expansion corresponds to intercalation of one layer of amide molecules with the orientation parallel to GO sheets. The high-temperature phase of BGO–C1 (*d*(001) = 8.97 Å) should also be added to this group due to one-layer intercalation. The structure of BGO in all four smaller amides can be confidently explained by intercalation of a single disordered layer of solvent molecules.

In contrast, BGO immersed in C5 and C6 displayed nearly 2-fold larger interlayer spacing than the BGO–C4 sample, with interlayer distances expanding relative to the solvent free state by 12.18 Å and 13.53 Å, respectively (Table 1). Melting of C8 and C10 resulted in a formation of BGO–C8 and BGO–C10 solvate phases with an even larger lattice expansion by 17.2 Å and 21.22 Å, respectively. It can be concluded that the increase in *c*-lattice expansion of BGO immersed in amides with the number of carbon atoms above 5 correlates with the increase of the alkyl chain length of the amide molecule. Expansion of the BGO structure in these solvents cannot be explained by intercalation of one solvent layer.

Therefore, two alternative possibilities need to be considered: the first one is multilayered structures with molecules parallel to GO planes and the second alternative is the “stand-up” orientation of amide molecules relative to GO planes. Below, we discuss geometrical constraints provided by the length of amide molecules (for the stand-up orientation) and thickness of molecule layers (for the parallel orientation).

The length of amide molecules can be easily calculated based on bond lengths and angles. The thickness of the amide layer can be estimated assuming the diameter of the molecule along the alkyl chain (considering the molecules roughly as cylinders). This diameter remains the same as the length of amines increases and even the longer amides can be packed into layers with similar thickness. This simple geometrical consideration allows us to explain the experimentally observed swelling of BGO in short amides. The length of four shorter amide molecules increases (C1 ≈ 3.3 Å, C2 ≈ 4.7 Å, C3 ≈ 6.1 Å, C4 ≈ 7.3 Å), but the BGO interlayer distance remains almost the same, increasing slightly but still corresponding to the thickness of the molecular layer. Notably, the experimentally observed thickness of one layer (given by the Δ*d* value, Table 1) increases slightly with an increment of about 0.12 Å per increment of the alkyl chain by one carbon atom. If this trend is extrapolated to longer amide molecules, the thickness of one C10 layer should correspond to ∼4.4 Å. However, this value is valid only for the first layer intercalated into the BGO structure, while the next layers are likely to be larger in size due to the more disordered structure.

The expansion of the BGO *c*-lattice in longer amides C5, C6, C8, and C10 is 12.18, 13.53, 17.2, and 20.92 Å, respectively. Considering the thickness of one molecular layer, these lattice expansions would correspond to approximately 3 layers intercalation for C5 and C6 and ∼5 layers for the BGO–C10 structure. Multilayered intercalation was observed previously for BGO swelling in alcohols.^18^ For example, swelling of BGO in octanol was found to result in the formation of a four-layered structure at ambient temperature, with a transition into a five-layered structure at lower temperatures.^54^ Therefore, the possibility of multilayered intercalation in amides of similar length needs to be taken into account. However, the sorption of alcohols in multilayered BGO–alcohol systems showed a rather clear trend correlating the number of parallel layers with a step-like increase in the amount of the sorbed solvent.^54,56^ This trend is not found in BGO–amide systems (Table 1).

The stand-up orientation of intercalated amide molecules is another feasible alternative to explain the experimentally found relations between expansion of the GO *c*-lattice and composition of solid solvates calculated using DSC data. The expansion of BGO by the value corresponding to double the length of alcohol molecules have been reported earlier for BGO in long (C > 10) alcohols.^46^ The experimentally observed expansion of BGO interlayer distance due to saturated swelling in longer (C5–C10) amides is too large to be explained by a single layer of solvent molecules inserted perpendicular to the GO basal planes and too small for a double layer. However, good correlation is found between the value of lattice expansion in BGO solvate structures with C5, C6, and C10 and the *c*-unit cell parameter of crystal structures of pure amides, which corresponds to a double layer of amide molecules but in a titled (rather than perpendicular) orientation relative to GO sheets (Table 2 and Figure 5).^66^ For example, the length of C10 amide is about 14 Å (and 28 Å double length), while the expansion of BGO in molten C10 is 21.22 Å. The *c*-unit cell parameter of solid C10 is 21.23 Å,^66^ in perfect agreement with BGO lattice expansion.

**Table 2:** Unit Cell Parameters in the Structure of Pure Amides (Monoclinic Structure of Similar Type, Except for Acetamide, Which Has an Orthorhombic Structure)^66^ Compared to the Expansion of Interlayer Distance of BGO Due to the Swelling Δ*d*(Å)^a^

Compound	Unit cell parameter *c* (Å)	Δ*d*(Å) BGO
Formamide (C1)	6.87	2.97 (RT); 6.32 (LT)
Acetamide (C2)	9.51 (orth)	3.48
Propionamide(C3)	8.97	3.59
Butyramide(C4)	10.02	3.72
Pentanamide(C5)	11.08	12.48
Hexanamide(C6)	14.11	13.83
Octanamide(C8)	17.39	17.20
Decanamide(C10)	21.23	21.22

a Both room-temperature (RT) and low-temperature (LT) values of Δ*d*(Å) phases are included for the BGO–formamide system. Similarity of Δ*d*(Å) with the *c*-parameter is found for C5–C10 structures.

**Figure 5:**
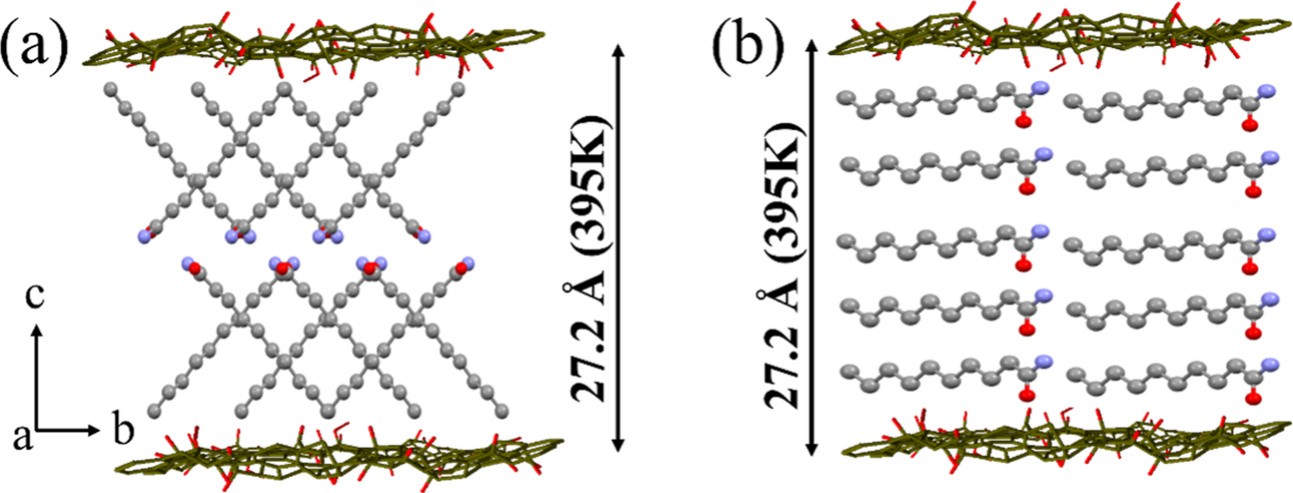
Suggestive structure of BGO intercalated by C10. The scheme illustrates the perfect match between expansion of the BGO lattice in molten decanamide and the *c*-unit cell parameter of the pure decanamide structure. (a) The scheme provides a view of BGO intercalated by decanamide with a tilted orientation corresponding to one unit cell of pure C10 structure inserted between GO sheets. (b) Hypothetical scheme of BGO intercalated by 5 layers of decanamide molecules parallel to GO planes.

Therefore, the arrangement of amide molecules confined between graphene oxide is in agreement with insertion of one unit cell fragment of the solid-state structure of amides (Figure 5a). It consists of two layers of amide molecules arranged head-to-head in a tilted orientation between the GO sheets, as illustrated using an example of the C10 structure in Figure 5a.^66^ The absence of XRD reflections due to intercalated solvents points to complete disorder of amide molecules, which is likely related to the inhomogeneous nature of GO itself. The model then accounts only for the orientation of molecules attached to GO sheets, with complete disorder of the structure along other directions.

The model shown in Figure 5a is also consistent with the composition of BGO–amides in the set C5 to C10, which remains almost unchanged in mol/g units. Moreover, the increment in *d*(001) values observed in BGO–CX (X = 5–10) (∼1.4 Å per one C atom length increase) is in good agreement with the increment of alkyl amide molecule length due to the addition of carbon atoms.

Further experiments aimed at the verification of parallel layered (Figure 5b) vs “stand-up” (Figure 5a) structural models showed results that are difficult to explain by parallel layer intercalation. The main evidence of multilayered intercalation of alkyl alcohols into the BGO structure presented in earlier publications relied on layer-by-layer evaporation of solvents during simple heating and vacuum annealing.^54,56^

Therefore, we performed additional experiments aimed at revealing the presence or absence of layered amide intercalation using in situ XRD experiments and the TGA method. The BGO–C8 system was selected for a more detailed XRD study due to a detailed earlier study available for BGO swelling in octanol (alcohol with 8 carbon atoms)^54^ and revealed structural changes compatible with layer-by-layer removal of C8 amide (Figure 6).

**Figure 6:**
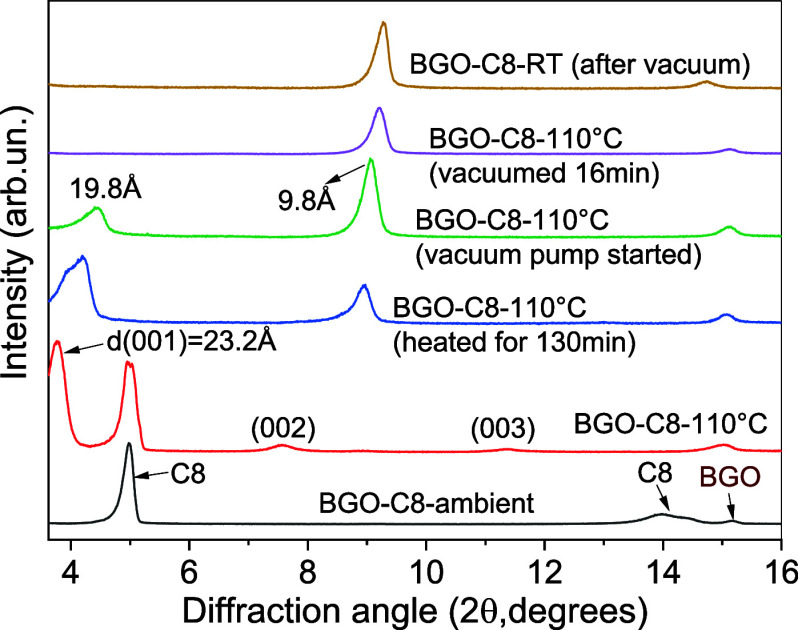
XRD patterns recorded from the BGO–C8 sample heated above the melting point of amide to allow swelling and maintained at 110 °C to allow evaporation of solvent. Since evaporation was too slow, vacuum pumping was added at a later annealing step (CuKα λ = 1.5418 Å).

The *d*(001) = 23.2 Å was found for BGO in the saturated liquid-immersed swelling state. Prolonged annealing of the BGO–C8 sample resulted first in evaporation of solvent excess and at the next stage in evolvement of a new phase with a *d*(001) value of 19.8­(Å). Evaporation rate for this phase appeared to be rather slow, and dynamic vacuum was added to accelerate the process. The system showed rapid loss of intercalated solvent with the formation of a new phase with *d*(001) = 9.8 Å, which can be confidently assigned to the formation of the BGO phase with one intercalated layer of C8. Therefore, this experiment can be interpreted as layer-by-layer evaporation starting from the 4L-BGO structure (23.2 Å) with a transition into the 3L-BGO structure (19.8 Å) and finally the 1L structure (9.8 Å). In absence of layered inetrcalation, evaporation of C8 would be expected to result in some gradual decrease of interlayer distance, possibly with the formation of the 1L-phase when the distance between neighboring molecules becomes large enough to allow the parallel to GO sheets orientation. Indeed, the change from 23.2 Å to 19.8 Å does not occur in one step and shows some additional shoulder (∼22.5 in the pattern shown in Figure 6). Also, no other intermediate phases, such as the 2L structure (expected at ∼14 Å), were discovered in the experiment shown in Figure 6.

The presence of some intermediate phases formed by desorption of solvent from saturated BGO–C8 was detected also using the TGA method. In this type of experiment, powder samples of BGO/amide were prepared using proportion corresponding to saturated swelling (sorption values in Table 1). The samples were heated in a TGA cell over the melting point of solvent and annealed at a constant temperature of 110 °C.

The TGA trace recorded from the BGO–C8 sample showed distinctly different weight loss rates, which show the existence of distinct phases (Figure 7a). Similar steps have been observed earlier in BGO–alcohol systems and explained by step-like desorption of solvent from a layered solvate structure.^61^

**Figure 7:**
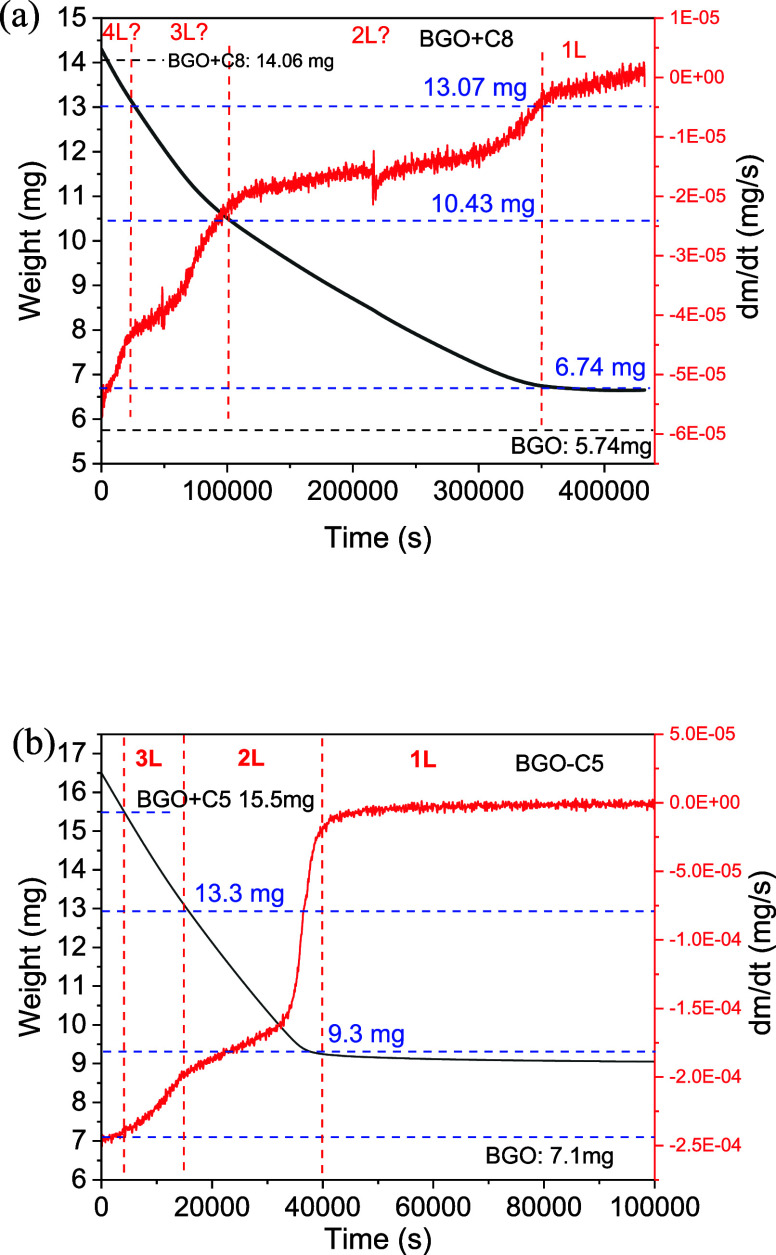
TGA scans showing weight loss curves (black) and weight loss rates (red) for BGO–C8 (a) and BGO–C5 (b) systems.

However, TGA data recorded from BGO–amides do not show weight changes by the same amount in each step, as expected for the sequential removal of equally dense solvent layers. Assuming that the steps in evaporation of C8 amide (Figure 7a) correspond hypothetically to removal of solvent layers, compositions of 1L, 2L, and 3L solvate phases would be 0.17 g/g, 0.82 g/g, and 1.28 g/g, respectively, with that of saturated 4L phase being 1.45 g/g. Notably, desorption of alcohols from multilayered BGO solvates was found in earlier studies to occur layer-by-layer, with weight loss steps approximately equal for each layer.^61^ Unequal amounts of solvent being desorbed at each desorption step points to the absence of a layered structure in BGO–C8. This conclusion is also in agreement with the XRD data. A similar result was also obtained in the TGA experiment with the BGO–C5 system, which also shows three steps in the evaporation of solvent, which could hypothetically be interpreted as a transition from the 3L phase to 2L and to 1L as the weight changes observed in these steps are not equal.

A detailed explanation of step-by-step changes of BGO–amide structures is not possible using available XRD and TGA data and is out of the scope of this study. However, analysis of available data allows us to rule out multilayered intercalation of C5 to C10 alkyl amides in the BGO structure.

## 4 Discussion

It is interesting to compare the swelling of BGO in alkyl amides with swelling in 1-alcohols of the same length (Figure 8). Both alcohols and amides consist of a polar end group and a nonpolar alkyl chain. Therefore, longer molecules are more hydrophobic, and short molecules are hydrophilic. The main difference is the polarity of the amide and alcohol end groups. Higher melting points of primary amides are related to stronger amide–amide interactions in the crystal structure of amides where the groups face each other. Therefore, one could expect some similarities in swelling related to the same chain structure of these molecules and also some differences due to stronger interactions between amide groups. The similarity between alkyl alcohols and alkyl amides includes not only the length of molecules with similar numbers of carbon atoms but also the thickness of one molecular layer intercalated between GO sheets.

**Figure 8:**
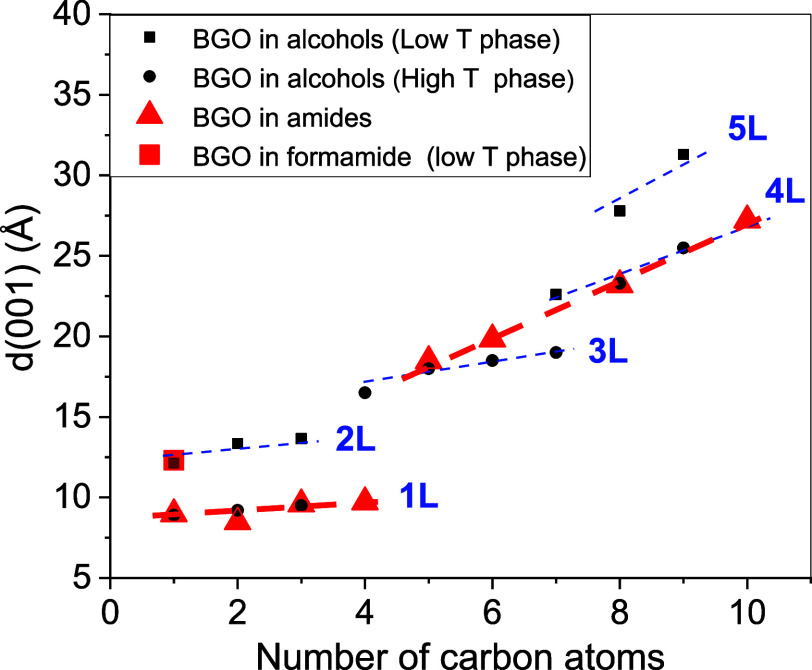
Interlayer distance of BGO–alcohols at low temperature (below the point of swelling transition) (■) and BGO–amides at just above the melting point of amide (■(red square)). Interlayer distance of BGO–alcohols at high temperature (above the point of swelling transition) (●) and BGO–amides near the melting point of amides (▲) (for C1 the interlayer distance of the high-temperature phase at ambient temperature). The values of interlayer distance of BGO in liquid alcohols are from our previous work.^44^

The main difference revealed by our experiments is the absence of 2L solvate phases in BGO–C2, BGO–C3, and BGO–C4 amide systems at near melting point temperatures (Figure 8) and absence of swelling transitions between 2L and 1L phases. All three amide solvents intercalate BGO only with one solvent layer. In contrast, BGO is intercalated with two layers of ethanol, two layers of propanol, and 3 layers of butanol near melting points of solvents. One could possibly assume that the melting points of amides (which are higher by tens of degrees than that of each corresponding alcohol) are simply “above” the possible points of swelling transitions in BGO–amide systems. The difference also extends to systems with longer alkyl chains. As described above, the structure of BGO–amide solvate phases (in the saturated swelling state) is unlikely to be layered. At the same time, there is clear evidence for 3–4–5 layered structures in BGO–alcohol systems, with swelling transitions between 5L and 4L structures found in BGO–heptanol, BGO–octanol, and BGO–nonanol systems.

It is interesting also to compare the composition of BGO solvate phases for amides and alcohols with similar lengths of alkyl chains. Figure 9 shows the amount of amide and alcohol adsorbed by BGO as a function of the carbon number in the molecular chain. For simplicity, the graph shows only high-temperature phases of BGO in alcohols for the systems where swelling transitions are known. Therefore, compositions of 1L solvate structures of BGO–alcohols are compared in this figure with 1L structures of BGO–amides. For longer alcohols, 3L and 4L structures are compared then with BGO–amide phases where solvent molecules are in the stand-up orientation (as in Figure 5).

**Figure 9:**
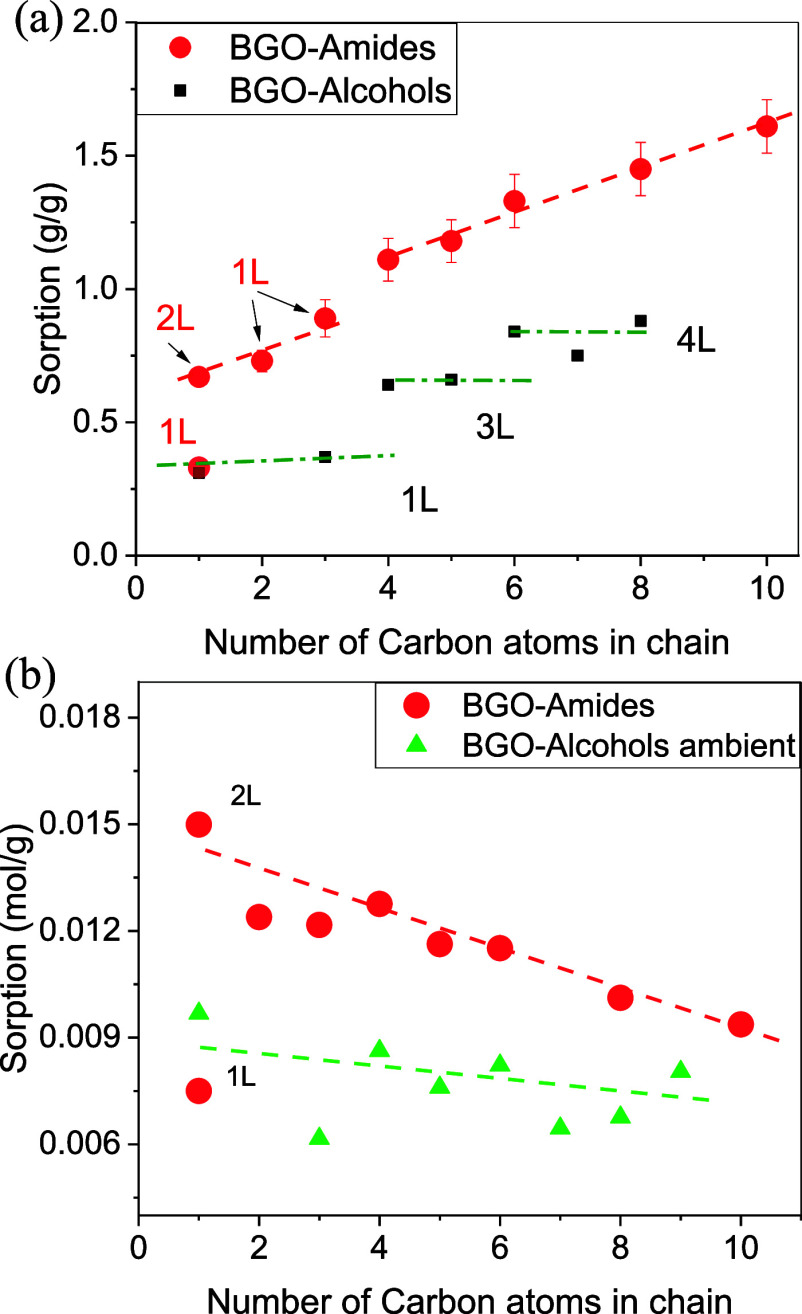
Compositions of BGO solvate phases in (a) g/g and (b) mol/g units with alkyl amides (this study) and alcohols; refs 41, 43, 56, and 67 For alcohols, the sorption values have been measured using vapor sorption (isopiestic method), while for amides, the values have been measured the using DSC method since the melting temperature points of amides are much higher than those of alcohols. For the BGO–formamide system, the value of sorption provided by DSC is shown for the 2L phase and a 2 times smaller value is shown for the 1L phase.

It should be noted that less clear trend and the stronger scatter of points for BGO–alcohol systems in the Figure 9b is explained partly by the absence of one study that included the same BGO material and exactly reproduced procedures. Instead, several studies have been published over the past decade, first for short alcohols and later for several longer alcohols (up to nonanol).^41,56,67^ Somewhat different BGOs with different oxidation degrees were used in these studies, also synthesized in one oxidation step or two oxidation steps.

Nevertheless, common trends can be established for both sorption of amides and sorption of alcohols. The sorption of amides increases approximately linearly in g/g units and slightly decreases in mol/g units along with the increase in the molecule length. The same trend is also found for BGO in alcohols with approximately similar molar sorption (in the range 0.006 mol/g–0.01 mol/g) and increased g/g sorption due to longer alkyl chains.

The only exception is BGO in formamide, which shows two times smaller sorption in g/g as compared to the trend observed for all other amides. In fact, the sorption value (mol/g) found for amide in the 1L phase is close to the value found for the 1L phase of BGO–methanol.^43^ Note that the molar mass of formamide is about 1.4 times higher than the molar mass of methanol. Nevertheless, it is obvious that the 1L solvate of BGO in formamide is much less dense than 1L phases of BGO in C2, C3, and C4 amides. Therefore, it can be hypothesized that the existence of the swelling transition found in the BGO–formamide system at low temperatures could possibly be related to the low density of solvent in the one-layered solvate structure. The amount of formamide sorbed by BGO to form the 2L structure is in good correlation with the overall trend observed for other BGO–amide materials.

In general, sorption of alcohols in high-temperature phases appears to be significantly smaller in mol/g units if compared to sorption of amides, indicating a strong difference in the structure of solvate phases. Once again, higher melting points of amides are likely to affect sorption of these solvents as these are liquids at higher temperatures (see melting points in Table 1), as compared to sorption data recorded for BGO in alcohols (Figure 9) at near ambient temperatures.

Comparing trends in the sorption of amides and alcohols vs length of alkyl chains provides one more argument against layered intercalation of amides in BGO structures in C5 to C10.

## 5 Conclusions

Swelling of BGO in several primary amides ranging from C1 to C10 has been studied using in situ synchrotron XRD and DSC as a function of temperature. The main results are as follows:

- BGO shows swelling in all of the molten amides studied here.
- The reversible Type I swelling transition found in the BGO–formamide system corresponds to the change between one-layer and two-layer intercalation of formamide molecules and the change of interlayer distance of BGO by 3.35 Å. The enthalpy of this transition measured by DSC is 0.01 kJ/g (BGO).
- No swelling transitions were found for BGO in other molten amides as a function of temperature, including acetamide (c2), propionamide (C3), butyramide (C4), valeramide (C5), hexanamide (C6), octanamide (C8), and decanamide (C10). This is in contrast to BGO–alcohol systems where swelling transitions have been reported for C2, C3, and C8.
- The sorption of all amides (except for formamide) by BGO near the point of solvent melting is similar (approximately 0.01 mol g^–1^), but a rather different expansion of the *c*-lattice is found by XRD.
- Swelling of BGO in molten acetamide, propionamide, and butyramide resulted in a *c*-lattice expansion by ∼3.5–3.7 Å corresponding to the formation of a structure with a single layer of amide molecules intercalated parallel to the GO planes.
- Intercalation of longer amides resulted in significantly larger expansion of the BGO interlayer distance correlating with the *c*-unit cell parameters of pure solid amides. Therefore, the swollen structure of BGO observed in molten valeramide, hexanamide, and decanamide is suggested to include two layers of amide molecules in a tilted “stand-up” orientation relative to the GO sheets.

Our study provides valuable fundamental insights into the swelling properties of BGO in alkyl amides featuring wide-range tunable interlayer distances and distinct swelling transitions. Precisely controlling the interlayer spacing of GO and the intercalation of different molecules between the GO layers have great potential to tune and improve the applications of GO such as element separation by sorption and others.^68^

## Supplementary Material



## Notes

Additional synchrotron XRD data and DSC plots for all the BGO–amide systems (PDF)

## References

[1] Brodie B. C. (1859). XIII On
the atomic weight of graphite. Philos. Trans.
R. Soc. London.

[2] Hummers W. S., Offeman R. E. (1958). Preparation
of graphitic oxide. J. Am. Chem. Soc..

[3] Marcano D. C., Kosynkin D. V., Berlin J. M., Sinitskii A., Sun Z., Slesarev A., Alemany L. B., Lu W., Tour J. M. (2010). Improved
Synthesis of Graphene Oxide. ACS Nano.

[4] Zaaba N. I., Foo K. L., Hashim U., Tan S. J., Liu W.-W., Voon C. H. (2017). Synthesis of Graphene
Oxide using Modified Hummers
Method: Solvent Influence. Procedia Eng..

[5] Chen J., Yao B., Li C., Shi G. (2013). An improved Hummers method for eco-friendly
synthesis of graphene oxide. Carbon.

[6] Gurzęda B., Boulanger N., Jørgensen M. R.
V., Kantor I., Talyzin A. V. (2024). Graphite oxide by “chlorate route” oxidation
without HNO3: Does acid matter?. Carbon.

[7] Gurzęda B., Florczak P., Kempiński M., Peplińska B., Krawczyk P., Jurga S. (2016). Synthesis of graphite
oxide by electrochemical
oxidation in aqueous perchloric acid. Carbon.

[8] Staudenmaier L. (1898). Verfahren
zur darstellung der graphitsäure. Ber.
Dtsch. Chem. Ges..

[9] Hofmann U., Frenzel A., Csalán E. (1934). Die Konstitution
der Graphitsäure
und ihre Reaktionen. Justus Liebigs Ann. Chem..

[10] Beck F., Jiang J., Krohn H. (1995). Potential
oscillations during galvanostatic
overoxidation of graphite in aqueous sulphuric acids. J. Electroanal. Chem..

[11] Lerf A., He H. Y., Forster M., Klinowski J. (1998). Structure
of graphite oxide revisited. J. Phys. Chem.
B.

[12] He H. Y., Klinowski J., Forster M., Lerf A. (1998). A new structural model
for graphite oxide. Chem. Phys. Lett..

[13] Szabo T., Berkesi O., Forgo P., Josepovits K., Sanakis Y., Petridis D., Dekany I. (2006). Evolution
of surface
functional groups in a series of progressively oxidized graphite oxides. Chem. Mater..

[14] Chua C. K., Sofer Z., Pumera M. (2012). Graphite Oxides: Effects of Permanganate
and Chlorate Oxidants on the Oxygen Composition. Chem—Eur. J..

[15] Eng A. Y. S., Chua C. K., Pumera M. (2015). Refinements to the structure of graphite
oxide: absolute quantification of functional groups via selective
labelling. Nanoscale.

[16] You S. J., Luzan S. M., Szabo T., Talyzin A. V. (2013). Effect of synthesis
method on solvation and exfoliation of graphite oxide. Carbon.

[17] Talyzin A. V., Mercier G., Klechikov A., Hedenström M., Johnels D., Wei D., Cotton D., Opitz A., Moons E. (2017). Brodie vs Hummers graphite oxides
for preparation of multi-layered
materials. Carbon.

[18] Iakunkov A., Talyzin A. V. (2020). Swelling properties of graphite oxides
and graphene
oxide multilayered materials. Nanoscale.

[19] Feicht P., Siegel R., Thurn H., Neubauer J. W., Seuss M., Szabó T., Talyzin A. V., Halbig C. E., Eigler S., Kunz D. A. (2017). Systematic evaluation of different types of graphene
oxide in respect to variations in their in-plane modulus. Carbon.

[20] Boehm H. P., Scholz W. (1965). Der “Verpuffungspunkt”—
des Graphitoxids. Z. Anorg. Allg. Chem..

[21] Korobov M. V., Talyzin A. V., Rebrikova A. T., Shilayeva E. A., Avramenko N. V., Gagarin A. N., Ferapontov N. B. (2016). Sorption
of polar organic solvents and water by graphite oxide: Thermodynamic
approach. Carbon.

[22] Yang E., Karahan H. E., Goh K., Chuah C. Y., Wang R., Bae T.-H. (2019). Scalable fabrication of graphene-based
laminate membranes
for liquid and gas separations by crosslinking-induced gelation and
doctor-blade casting. Carbon.

[23] Gao W., Majumder M., Alemany L. B., Narayanan T. N., Ibarra M. A., Pradhan B. K., Ajayan P. M. (2011). Engineered Graphite
Oxide Materials for Application in Water Purification. ACS Appl. Mater. Interfaces.

[24] Yuan Y., Gao X., Wei Y., Wang X., Wang J., Zhang Y., Gao C. (2017). Enhanced desalination performance of carboxyl functionalized graphene
oxide nanofiltration membranes. Desalination.

[25] Kumar R., Joanni E., Savu R., Pereira M. S., Singh R. K., Constantino C. J. L., Kubota L. T., Matsuda A., Moshkalev S. A. (2019). Fabrication
and electrochemical evaluation of micro-supercapacitors prepared by
direct laser writing on free-standing graphite oxide paper. Energy.

[26] Tian Y., Yu Z., Cao L., Zhang X. L., Sun C., Wang D.-W. (2021). Graphene
oxide: An emerging electromaterial for energy storage and conversion. J. Energy Chem..

[27] Sun J., Iakunkov A., Rebrikova A. T., Talyzin A. V. (2018). Exactly matched
pore size for the intercalation of electrolyte ions determined using
the tunable swelling of graphite oxide in supercapacitor electrodes. Nanoscale.

[28] Zhang P. X., Wang Q., Zhang Y. X., Lin M., Zhou X., David A., Ustyuzhanin A., Chen M. S., Katsnelson M. I., Trubyanov M. (2025). Strain-induced crumpling of graphene oxide
lamellas to achieve fast and selective transport of H and CO. Nat. Nanotechnol..

[29] Derksen J. C., Katz J. R. (1934). Untersuchungen über
die Intramicellare Quellung
der Graphitsäure. Erste Mitteilung. Isotherme. Einfluss von
lyotropen Substanzen, von Temperatur und von PH auf das Quellungsmaximum. Recl. Trav. Chim. Pays-Bas.

[30] Ruiz J. C., Macewan D. M. C. (1955). Interlamellar Sorption Complexes
of Graphitic Acid
with Organic Substances. Nature.

[31] AragÓN F., Cano Ruiz J., Macewan D. M. C. (1959). β-Type Interlamellar Sorption
Complexes. Nature.

[32] Boehm H. P., Clauss A., Hofmann U. (1961). Graphite Oxide and Its Membrane Properties. J. Chim Phys. Pcb.

[33] Talyzin A. V., Hausmaninger T., You S. J., Szabo T. (2014). The structure of graphene
oxide membranes in liquid water, ethanol and water-ethanol mixtures. Nanoscale.

[34] Iakunkov A., Sun J., Rebrikova A., Korobov M., Klechikov A., Vorobiev A., Boulanger N., Talyzin A. V. (2019). Swelling of graphene
oxide membranes in alcohols: effects of molecule size and air ageing. J. Mater. Chem. A.

[35] Cerveny S., Barroso-Bujans F., Alegría A. ´., Colmenero J. (2010). Dynamics of
Water Intercalated in Graphite Oxide. J. Phys.
Chem. C.

[36] Lerf A., Buchsteiner A., Pieper J., Schottl S., Dekany I., Szabo T., Boehm H. P. (2006). Hydration behavior and dynamics of
water molecules in graphite oxide. J. Phys.
Chem. Solids.

[37] Klechikov A., You S., Lackner L., Sun J., Iakunkov A., Rebrikova A., Korobov M., Baburin I., Seifert G., Talyzin A. V. (2018). Graphite
oxide swelling in molten sugar alcohols and their aqueous solutions. Carbon.

[38] Zheng S., Tu Q., Wang M., Urban J. J., Mi B. (2020). Correlating Interlayer
Spacing and Separation Capability of Graphene Oxide Membranes in Organic
Solvents. ACS Nano.

[39] Xiao P., Xiao M., Liu P., Gong K. (2000). Direct synthesis
of
a polyaniline-intercalated graphite oxide nanocomposite. Carbon.

[40] Klechikov A., Yu J., Thomas D., Sharifi T., Talyzin A. V. (2015). Structure of graphene
oxide membranes in solvents and solutions. Nanoscale.

[41] Barroso-Bujans F., Cerveny S., Alegría A., Colmenero J. (2010). Sorption and
desorption behavior of water and organic solvents from graphite oxide. Carbon.

[42] Li G., Gurzęda B., Iakunkov A., Nordenström A., Boulanger N., Hennig C., Dejoie C., Talyzin A. V. (2025). Temperature
Dependent Swelling Transitions of Hummers Graphite Oxide in Liquid
1-Alcohols. Adv. Mater. Interfaces.

[43] You S., Luzan S., Yu J., Sundqvist B., Talyzin A. V. (2012). Phase Transitions in Graphite Oxide Solvates at Temperatures
Near Ambient. J. Phys. Chem. Lett..

[44] Iakunkov A., Nordenström A., Boulanger N., Li G., Hennig C., Jørgensen M. R. V., Kantor I., Talyzin A. V. (2024). Effect of Chain
Length on Swelling Transitions of Brodie Graphite Oxide in Liquid
1-Alcohols. Adv. Mater. Interfaces.

[45] Aragon F., Ruiz J. C., Macewan D. M. C. (1959). Beta-Type Interlamellar
Sorption
Complexes. Nature.

[46] Pino C. D., Ramirez A., Cano-ruiz J. (1966). Measurement of Graphitic Acid Surface
with Polar Molecules. Nature.

[47] Garcia A. R., Canoruiz J., Macewan D. M. C. (1964). Temperature Variation of Beta-Type
Interlamellar Sorption Complexes of Graphitic Acid with Alcohols. Nature.

[48] Talyzin A. V., Solozhenko V. L., Kurakevych O. O., Szabó T., Dékány I., Kurnosov A., Dmitriev V. (2008). Colossal Pressure-Induced
Lattice Expansion of Graphite Oxide in the Presence of Water. Angew. Chem. Int. Edit.

[49] Talyzin A. V., Sundqvist B., Szabo T., Dekany I., Dmitriev V. (2009). Pressure-Induced
Insertion of Liquid Alcohols into Graphite Oxide Structure. J. Am. Chem. Soc..

[50] Talyzin A. V., Luzan S. M. (2010). Pressure-Induced Insertion of Liquid Acetone into the
Graphite Oxide Structure. J. Phys. Chem. C.

[51] Boulanger N., Skrypnychuk V., Nordenström A., Moreno-Fernández G., Granados-Moreno M., Carriazo D., Mysyk R., Bracciale G., Bondavalli P., Talyzin A. V. (2021). Spray Deposition of Supercapacitor
Electrodes using Environmentally Friendly Aqueous Activated Graphene
and Activated Carbon Dispersions for Industrial Implementation. ChemElectroChem.

[52] Cabrillo C., Barroso-Bujans F., Fernandez-Perea R., Fernandez-Alonso F., Bowron D., Bermejo F. J. (2016). Absorbate-induced ordering and bilayer
formation in propanol-graphite-oxide intercalates. Carbon.

[53] Lin H., Iakunkov A., Severin N., Talyzin A. V., Rabe J. P. (2022). Rapid Aging
of Bilayer Graphene Oxide. J. Phys. Chem. C.

[54] Klechikov A., Sun J., Baburin I. A., Seifert G., Rebrikova A. T., Avramenko N. V., Korobov M. V., Talyzin A. V. (2017). Multilayered intercalation
of 1-octanol into Brodie graphite oxide. Nanoscale.

[55] Talyzin A. V., Luzan S. M., Szabo T., Chernyshev D., Dmitriev V. (2011). Temperature dependent structural
breathing of hydrated
graphite oxide in H2O. Carbon.

[56] Rebrikova A. T., Klechikov A., Iakunkov A., Sun J., Talyzin A. V., Avramenko N. V., Korobov M. (2020). Swollen Structures of Brodie Graphite
Oxide as Solid Solvates. J. Phys. Chem. C.

[57] You S., Sundqvist B., Talyzin A. V. (2013). Enormous Lattice Expansion of Hummers
Graphite Oxide in Alcohols at Low Temperatures. ACS Nano.

[58] Nordenström A., Iakunkov A., Boulanger N., Li G., Hennig C., Baburin I., Jørgensen M., Kantor I., Talyzin A. V. (2023). Temperature
dependent intercalation of molten 1-hexadecanol into Brodie graphite
oxide. Carbon.

[59] Macewan D. M. C., De La Cruz F. A. (1959). Phase Transitions in Interlamellar
Films. Nature.

[60] Johansson, I. Amides, Fatty Acid. In Kirk-Othmer Encyclopedia of Chemical Technology; Wiley, 2003.

[61] Talyzin A. V., Solozhenko V. L., Kurakevych O. O., Szabó T., Dékány I., Kurnosov A., Dmitriev V. (2008). Colossal Pressure-Induced
Lattice Expansion of Graphite Oxide in the Presence of Water. Angew. Chem., Int. Ed..

[62] Ladell J., Post B. (1954). The crystal structure of formamide. Acta Crystallogr..

[63] Grabato, J. R. H. ; Federico, S. A. P. ; Hizon-Fradejas, A. B. ; Mojica, E.-R. E. Formamide. In Encyclopedia of Toxicology; Wiley, 2024; pp 831–835.

[64] Abate L., Badea E., Blanco I., Della Gatta G. (2008). Heat Capacities
and Enthalpies of Solid–Solid Transitions and Fusion of a Series
of Eleven Primary Alkylamides by Differential Scanning Calorimetry. J. Chem. Eng. Data.

[65] Ralston, A. W. Fatty acids and their derivatives; John Wiley & Sons, Inc., 1948.

[66] Turner J.
D., Lingafelter E. C. (1955). The X-Ray
Crystallography of the Normal-Aliphatic Amides. Acta Crystallogr..

[67] You S., Yu J., Sundqvist B., Belyaeva L. A., Avramenko N. V., Korobov M. V., Talyzin A. V. (2013). Selective Intercalation of Graphite
Oxide by Methanol in Water/Methanol Mixtures. J. Phys. Chem. C.

[68] Song Y., Li R., Pan F., He Z., Yang H., Li Y., Yang L., Wang M., Wang H., Jiang Z. (2019). Ultrapermeable
graphene oxide membranes with tunable interlayer distancesviavein-like
supramolecular dendrimers. J. Mater. Chem. A.

