# Supplementary material for: Temperature-Dependent Swelling of Brodie Graphite Oxide in Liquid Primary Amides
Source: J Phys Chem C Nanomater Interfaces. 2026 May 26;130(22):7874–85. doi: 10.1021/acs.jpcc.6c01991 (PMC13244798; doi:10.1021/acs.jpcc.6c01991)
Supplement: Supplementary file 1 [file jp6c01991_si_001.pdf]

Supporting Information

“Temperature-dependent swelling of Brodie graphite oxide in liquid primary amides”

Gui Li,[1] Nicolas Boulanger,[1] Bartosz Gurzęda,[2,3] Christoph Hennig,[2,3] Kristina Kvashnina,[2,3]

*Alexandr V Talyzin.[1]**

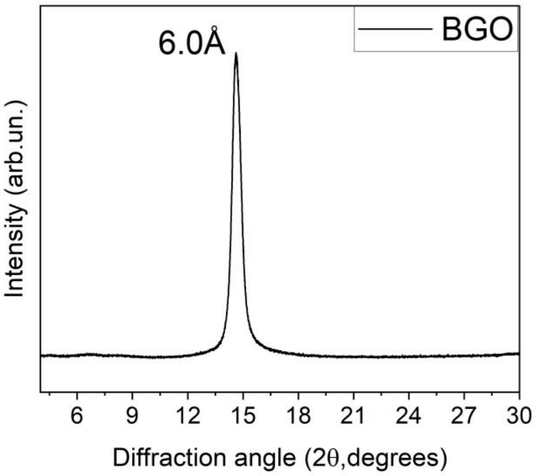

Figure S1. XRD pattern of pure BGO. (Cu Kα)

Table S1. Mass loading of BGO and Amides for XRD and DSC.

Systems	XRD		DSC	
	BGO	Amides	BGO	Amides
**BGO-C1**	-	-	11.6mg	13.0mg
**BGO-C2**	9.1mg	55.6mg	4.8mg	21.9mg
**BGO-C3**	11.6mg	57.0mg	2.8mg	16.6mg
**BGO-C4**	11.0mg	57.8mg	2.5mg	12.9mg
**BGO-C5**	9.7mg	51.5mg	2.7mg	13.5mg
**BGO-C6**	10.5mg	58.5mg	2.4mg	13.0mg
**BGO-C8**	-	-	2.4mg	13.2mg
**BGO-C10**	11.4mg	63mg	2.6mg	15.9mg

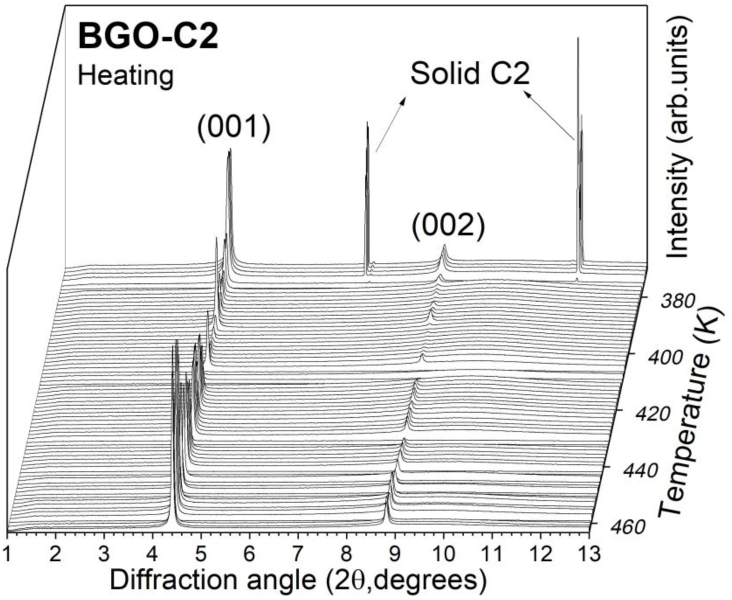

Figure S2. Temperature-dependent XRD patterns recorded from the BGO-C2 sample premixed as powders with excess of alcohol required for saturated swelling and heated over the temperature of C2 melting. (λ=0.727692Å)

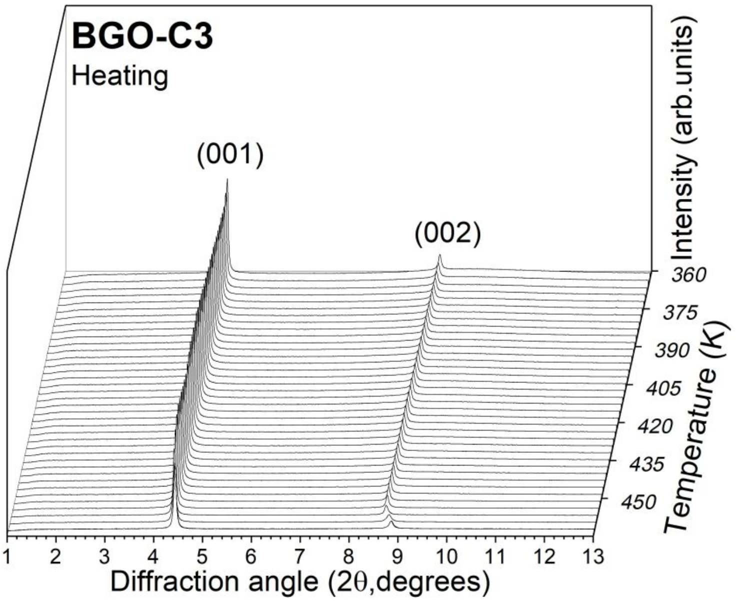

Figure S3. Temperature-dependent XRD patterns recorded from the BGO-C3 sample premixed as powders with excess of alcohol required for saturated swelling and heated over the temperature of C3 melting. (λ=0.727692Å)

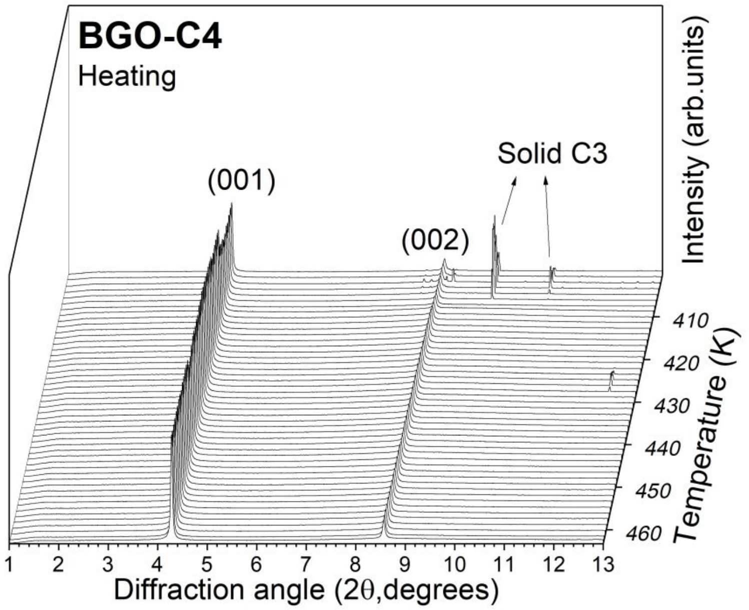

Figure S4. Temperature-dependent XRD patterns recorded from the BGO-C4 sample premixed as powders with excess of alcohol required for saturated swelling and heated over the temperature of C4 melting. (λ=0.727692Å)

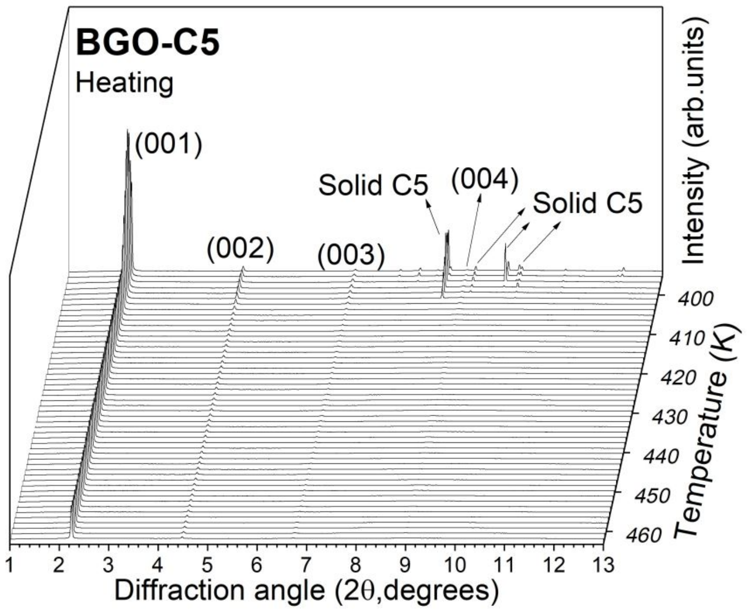

Figure S5. Temperature-dependent XRD patterns recorded from the BGO-C5 sample premixed as powders with excess of alcohol required for saturated swelling and heated over the temperature of C5 melting. (λ=0.727692Å)

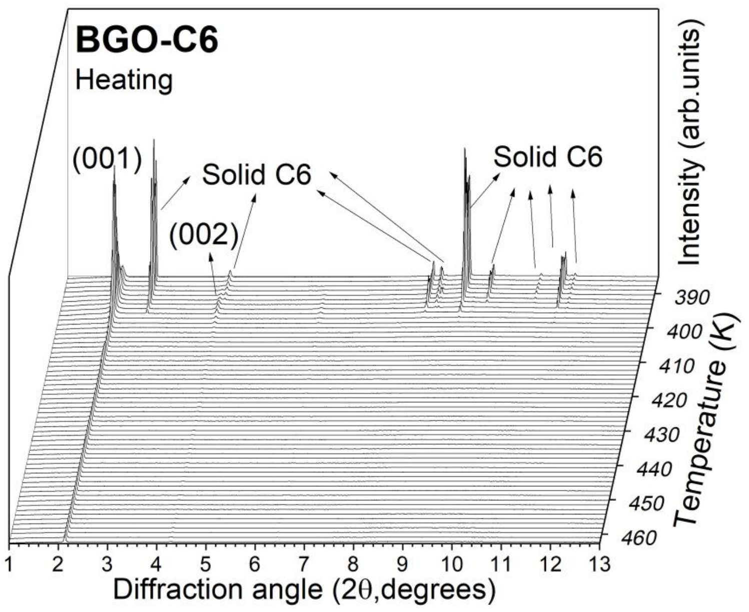

Figure S6. Temperature-dependent XRD patterns recorded from the BGO-C6 sample premixed as powders with excess of alcohol required for saturated swelling and heated over the temperature of C6 melting. (λ=0.727692Å)

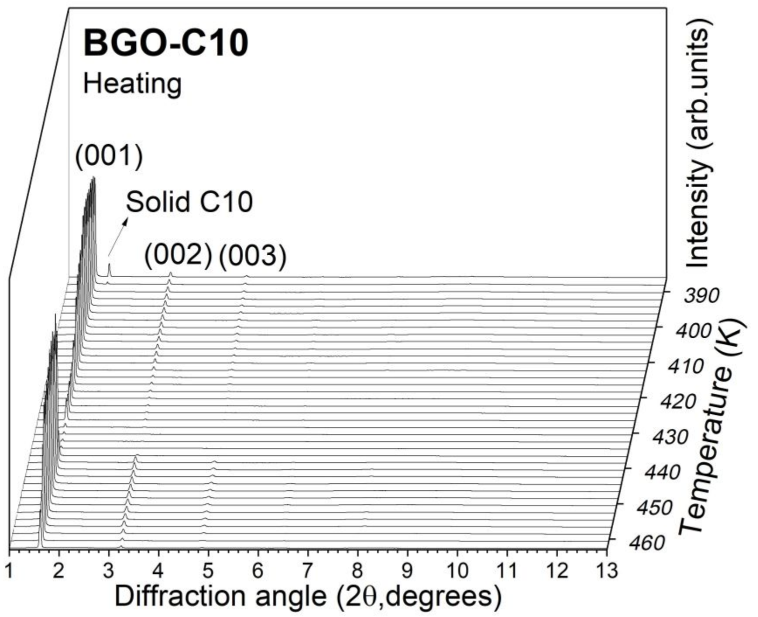

Figure S7. Temperature-dependent XRD patterns recorded from the BGO-C10 sample premixed as powders with excess of alcohol required for saturated swelling and heated over the temperature of C10 melting. (λ=0.727692Å)

DSC results

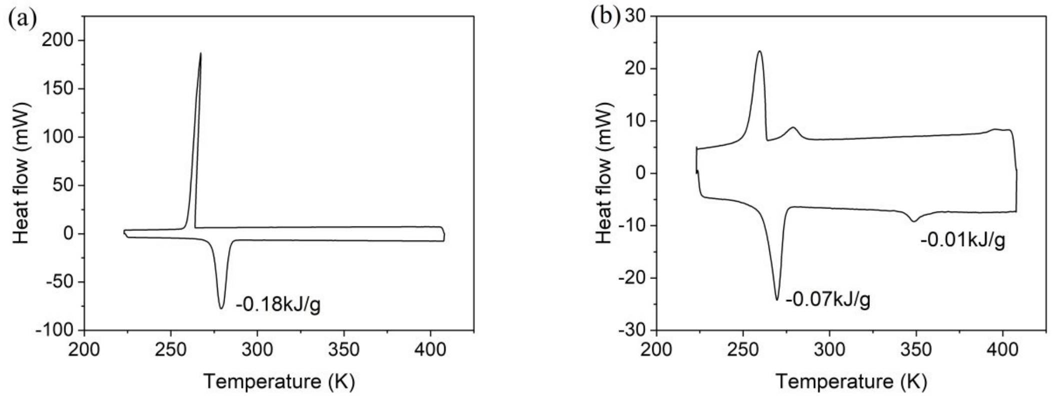

Figure S8. DSC traces of (a) C1, and (b) BGO/C1 mixture.

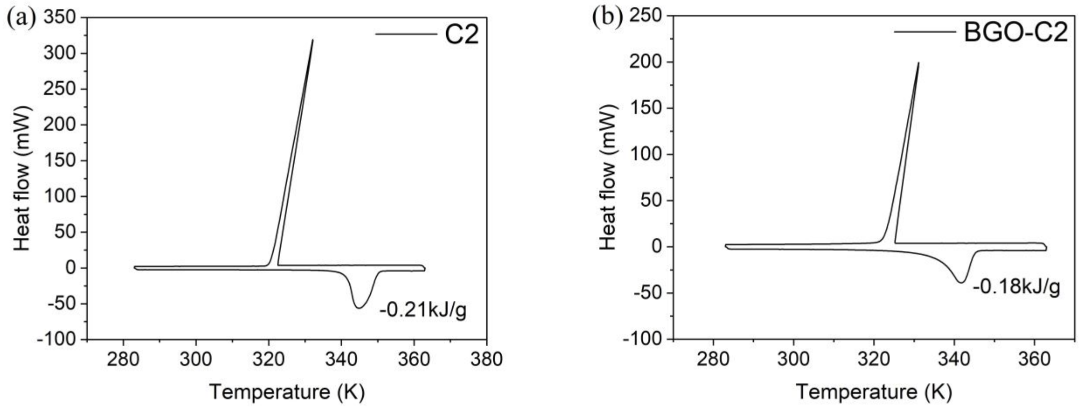

Figure S9. DSC traces of (a) C2, and (b) BGO/C2 mixture.

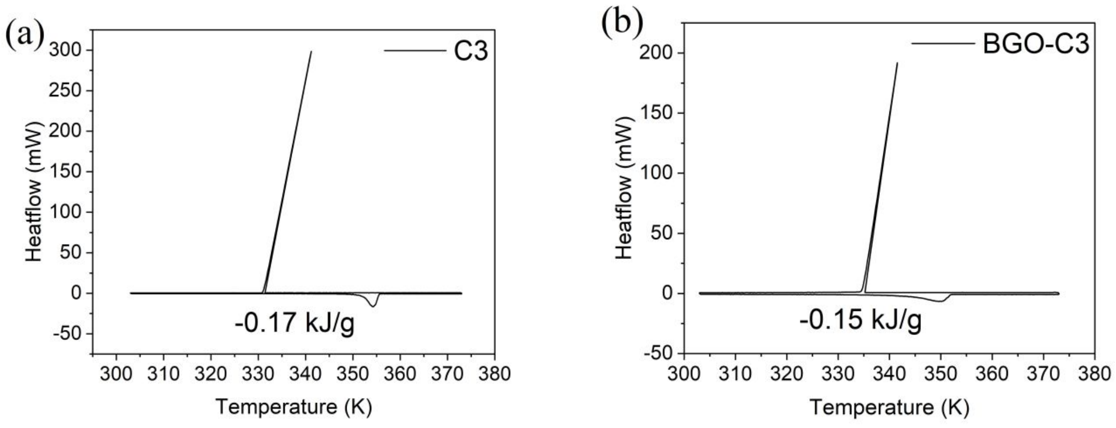

Figure S10. DSC traces of (a) C3, and (b) BGO/C3 mixture.

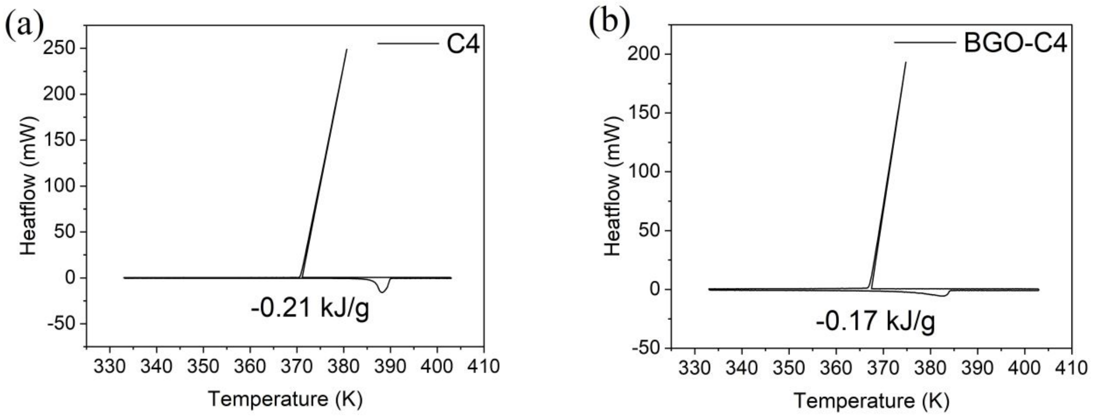

Figure S11. DSC traces of (a) C4, and (b) BGO/C4 mixture.

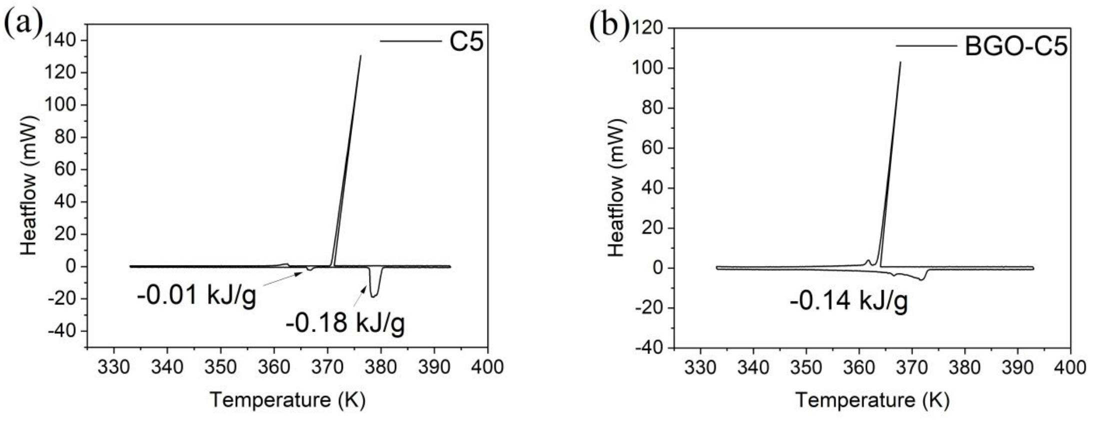

Figure S12. DSC traces of (a) C5, and (b) BGO/C5 mixture.

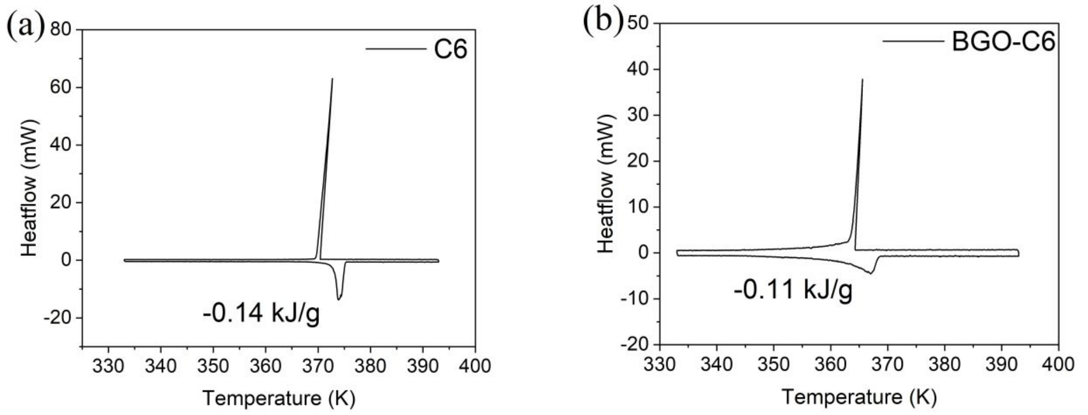

Figure S13. DSC traces of (a) C6, and (b) BGO/C6 mixture.

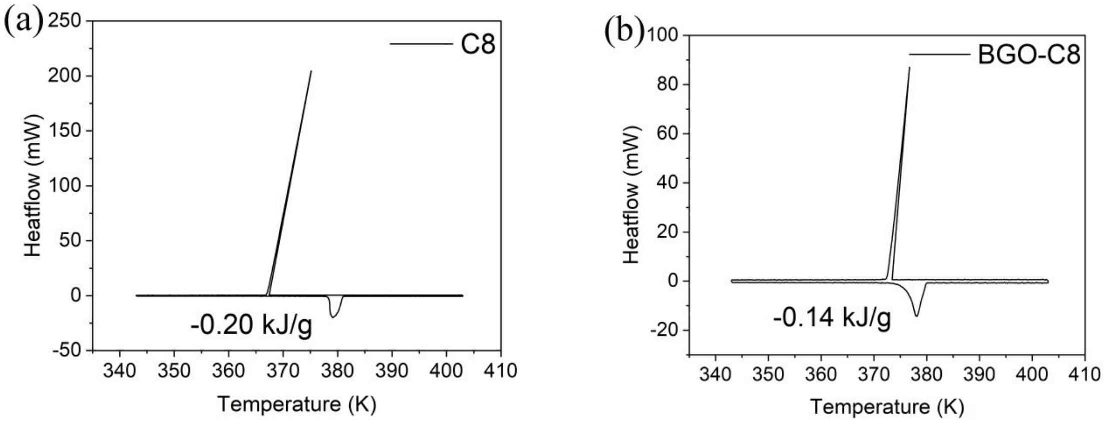

Figure S14. DSC traces of (a) C8, and (b) BGO/C8 mixture.

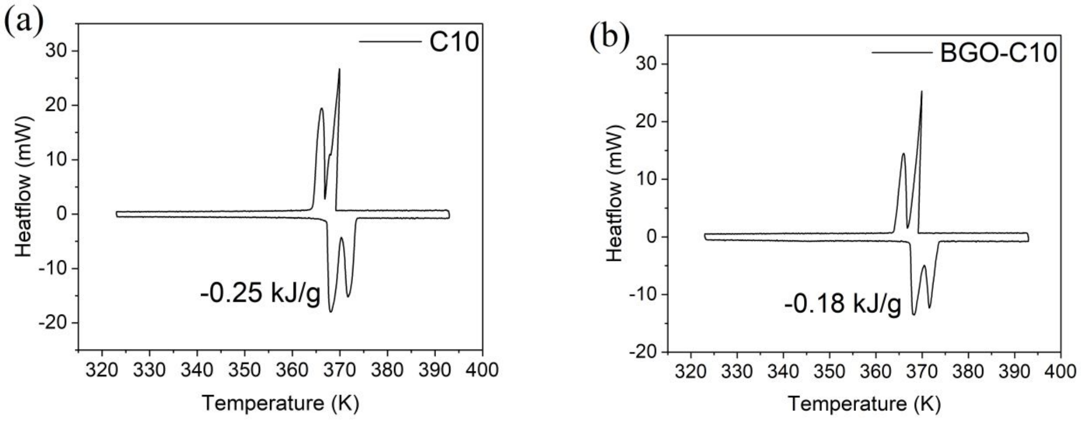

Figure S15. DSC traces of (a) C10, and (b) BGO/C10 mixture.

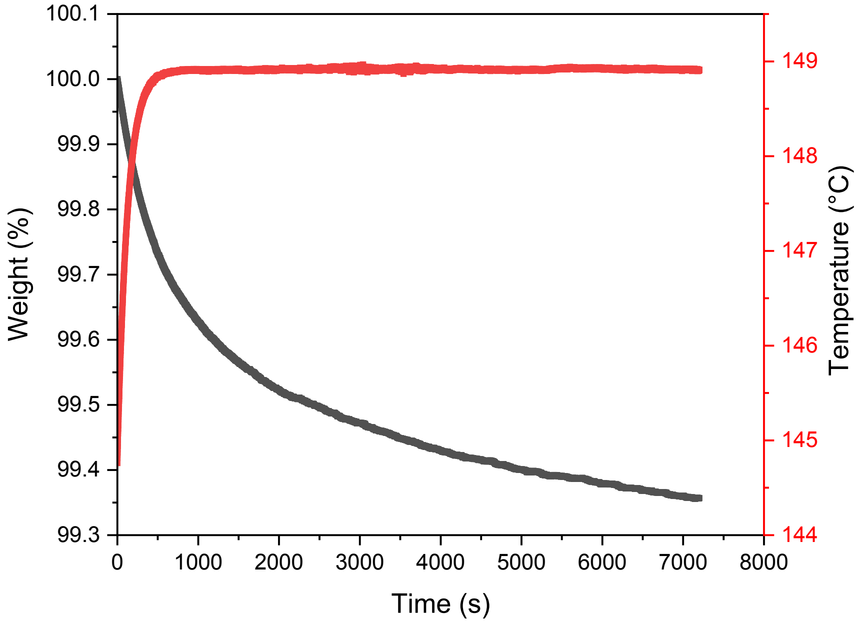

Figure S16 TGA trace of BGO recorded at stable temperature (149°C) over the period of 2 hours (similar to experiments with swelling in amides). The sample was first pre-heated in TGA at 110C to remove water sorbed from air (~5 wt% loss).

Standard TGA scans from the same batch of BGO were published in our earlier studies for full range of temperatures, see SI file Figure S2 in ref [1] and TGA scans of BGO plotted together with HGO in the Figure 5 ref. [6,]

(1) Iakunkov, A.; Nordenström, A.; Boulanger, N.; Li, G.; Hennig, C.; Jørgensen, M. R. V.; Kantor, I.; Talyzin, A. V. Effect of Chain Length on Swelling Transitions of Brodie Graphite Oxide in Liquid 1-Alcohols. *Advanced Materials Interfaces* **2023**, *11* (1). DOI: 10.1002/admi.202300554.
